# Prolonged activation of innate immune pathways by a polyvalent STING agonist

**DOI:** 10.1038/s41551-020-00675-9

**Published:** 2021-02-08

**Authors:** Suxin Li, Min Luo, Zhaohui Wang, Qiang Feng, Jonathan Wilhelm, Xu Wang, Wei Li, Jian Wang, Agnieszka Cholka, Yang-xin Fu, Baran D. Sumer, Hongtao Yu, Jinming Gao

**Affiliations:** 1 Department of Pharmacology, Harold C. Simmons Comprehensive Cancer Center, University of Texas Southwestern Medical Center, Dallas, TX USA; 2 Department of Pathology, Harold C. Simmons Comprehensive Cancer Center, University of Texas Southwestern Medical Center, Dallas, TX USA; 3 Department of Otolaryngology, Harold C. Simmons Comprehensive Cancer Center, University of Texas Southwestern Medical Center, Dallas, TX USA; 4 Howard Hughes Medical Institute, University of Texas Southwestern Medical Center, Dallas, TX USA; 5 Present Address: Zhejiang Provincial Laboratory of Life Sciences and Biomedicine, School of Life Sciences, Westlake University, Hangzhou, China

**Keywords:** Cancer, Cancer therapy, Biomaterials

## Abstract

The stimulator of interferon genes (STING) is an endoplasmic reticulum transmembrane protein that is a target of therapeutics for infectious diseases and cancer. However, early-phase clinical trials of small-molecule STING agonists have shown limited antitumour efficacy and dose-limiting toxicity. Here, we show that a polyvalent STING agonist—a pH-sensitive polymer bearing a seven-membered ring with a tertiary amine (PC7A)—activates innate-immunity pathways through the polymer-induced formation of STING–PC7A condensates. In contrast to the natural STING ligand 2′,3′-cyclic-GMP-AMP (cGAMP), PC7A stimulates the prolonged production of pro-inflammatory cytokines by binding to a non-competitive STING surface site that is distinct from the cGAMP binding pocket. PC7A induces antitumour responses that are dependent on STING expression and CD8^+^ T-cell activity, and the combination of PC7A and cGAMP led to synergistic therapeutic outcomes (including the activation of cGAMP-resistant STING variants) in mice bearing subcutaneous tumours and in resected human tumours and lymph nodes. The activation of the STING pathway through polymer-induced STING condensation may offer new therapeutic opportunities.

## Abstract (web summary)

A polyvalent STING agonist prolongs the activation of innate-immunity pathways through the formation of STING condensates, and leads to synergistic therapeutic outcomes in vivo when combined with the STING ligand cGAMP.

## Main

STING plays a central role in innate immunity against infection and cancer^1–4^. STING is endogenously activated by cGAMP, a cyclic dinucleotide that is synthesized by cGAMP synthase (cGAS) in response to cytosolic DNA as a danger signal^5,6^. Activation of STING mediates a multifaceted type-I interferon (IFN-I) response that promotes the maturation and migration of dendritic cells (DCs), and primes cytotoxic T lymphocytes and natural killer (NK) cells for spontaneous immune responses^7–11^. In recent years, STING has emerged as an important target that activates antitumour immune pathways for cancer immunotherapy^12–17^. Previous studies have observed punctate structures after the addition of cGAMP to STING, indicating that oligomerization or even higher-order architecture may be critical for activation^18–21^. Therapeutic attempts to deliver cGAMP into the cytosol of target cells in which STING is located have been limited by its inherent properties as a small, dual negatively charged molecule^22^. Moreover, the rapid enzymatic degradation and clearance as well as off-target toxicity of cGAMP have hindered its further clinical application^23,24^. Thus, the pharmaceutical industry has devoted great efforts to the chemical modification of natural cyclic dinucleotides (CDNs) as well as developing new STING agonists to improve their bioavailability and pharmacological activity^25,26^. Despite therapeutic promise, several small-molecule agonists of STING have shown limited antitumour efficacy and dose-limiting toxicity in early-phase clinical trials^27,28^.

Polyvalent phase condensation has been shown to regulate diverse biological processes, including ribosome assembly, gene expression and signal transduction^29,30^. Phase separation involves the assembly of macromolecular complexes through multivalent interactions^31^. A previous study has shown that DNA-induced liquid phase separation of cGAS triggers innate immunity^32^. By forming such biomolecular condensates, proteins involved in signalling cascades can be easily enriched in membraneless assemblies and amplify responses to small changes in the microenvironment. These biomolecular condensates are typically hundreds of nanometres to micrometres in size, and are transient and dynamic in response to specific stimuli or stress^33,34^.

We previously synthesized a library of pH-sensitive polymers with linear or cyclic tertiary amine structures, among which a polymer with a cyclic seven-membered ring (PC7A) has shown a strong vaccine adjuvant effect through the STING-dependent pathway^17^. In this Article, we report that PC7A is a polyvalent STING agonist that acts through polymer-induced phase condensation of STING to activate an innate immune response with prolonged cytokine expression compared with cGAMP. The level of STING activation depends on the length of the polymer and thereby the valency of the interaction. We also demonstrate that PC7A nanoparticles (NPs) loaded with cGAMP lead to robust tumour growth inhibition and enhanced survival in two animal tumour models, and synergistic STING activation in resected human tumours and lymph nodes. We provide a proof of principle for new cancer immunotherapy strategies targeting the STING pathway.

## Results

### PC7A polymer activates STING with a spatiotemporal profile that is distinct from cGAMP

**Fig. 1:**
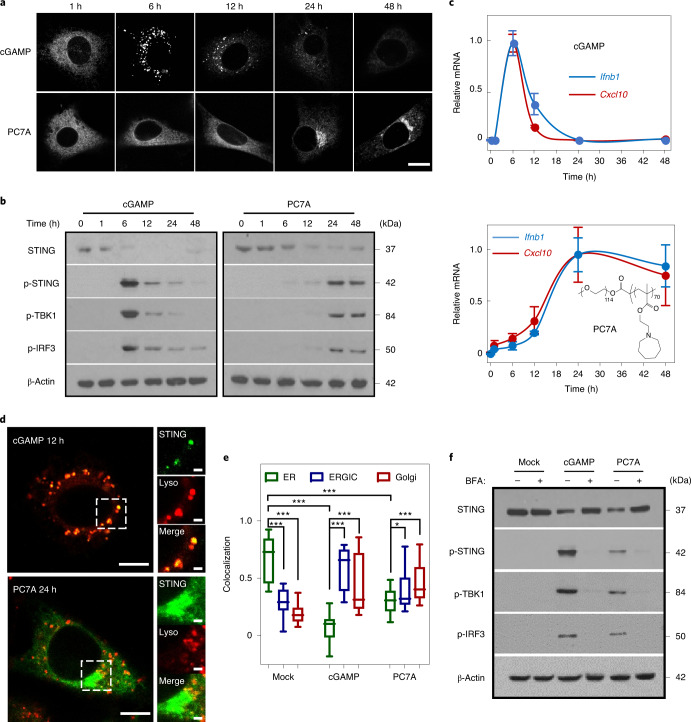
PC7A polymer activates STING with a spatiotemporal profile that is distinct from cGAMP. **a**, MEFs primed by cGAMP or PC7A exhibit different geometric and temporal patterns of GFP–STING-punctate formation and depletion. Cells were first incubated with cGAMP (10 μM, PEI was used for cytosolic delivery; Supplementary Fig. 1a,b) or PC7A micelles (10 μM) for 1 h, and the medium was then exchanged and cells were incubated for the indicated periods before imaging. Scale bar, 10 μm. **b**, THP1 cells that were treated with cGAMP display a burst effect of TBK1/IRF3 phosphorylation followed by rapid STING degradation, whereas treatment with PC7A led to sustained TBK1/IRF3 phosphorylation and slower STING degradation. **c**, Relative *Ifnb1* and *Cxcl10* mRNA levels show slower but prolonged STING activation in THP1 cells by PC7A compared with cGAMP. Data are mean ± s.d. *n* = 3 biologically independent experiments. **d**, GFP–STING colocalizes with lysosomes in MEFs 12 h after cGAMP treatment, supporting rapid degradation. By contrast, PC7A inhibits lysosomal degradation of GFP–STING, as indicated by the lack of colocalization and persistent GFP fluorescence. Scale bars, 5 μm (left images); 1 μm (right images). **e**, cGAMP and PC7A induce similar STING translocation from the ER to the ERGIC and Golgi apparatus. Colocalization was quantified using the Pearson’s correlation coefficient. For the box plot, the centre line is the mean, the box limits show the 25th to 75th percentile, and the whiskers show the minimum and maximum values. *n* = 20 cells examined over three independent experiments. Statistical analysis was performed using two-tailed Student’s *t*-tests (PC7A treatment group: ER versus ERGIC, *P = 0.029; ER versus Golgi, ***P = 0.0005; for all other comparisons, ***P < 0.0001). **f**, STING translocation is necessary for downstream signalling as BFA, which is an inhibitor of protein transport from ER to Golgi, prevents the phosphorylation of TBK1/IRF3 by cGAMP or PC7A. The confocal images in **a** and **d** are representative of at least three biologically independent experiments.

To understand how PC7A-induced STING activation differs from cGAMP^20,21^, we first investigated the intracellular distribution of GFP-labelled STING and the downstream signals in live cells in response to treatment. Remarkably, the temporal profile of PC7A-induced STING-puncta formation and maturation is distinct from those induced by cGAMP. When primed by cGAMP, STING-puncta formation occurs rapidly, producing a strong immune response that peaks around 6 h after stimulation, followed by rapid degradation and subsequent immune silence (Fig. 1a–c). By contrast, PC7A generates a durable STING activation profile, with sustained expression of interferon-stimulated genes (*Ifnb1* and *Cxcl10*) over 48 h. STING degradation is delayed after PC7A stimulation, as indicated by the limited fusion of STING-puncta with lysosomes even at 48 h (Fig. 1d and Supplementary Fig. 1c). We observed a similar effect of delayed STING degradation in cGAMP-treated cells that were preincubated with bafilomycin A1—a vacuolar H^+^ ATPase inhibitor that blocks lysosome acidification—and in cells treated with combined treatment of cGAMP and PC7A (Supplementary Fig. 1d,e). Overall, these data suggest that the endo-lysosomal pH-buffering ability of PC7A may be responsible for slow STING degradation^35^.

Despite the differences in size and kinetics of puncta formation, intracellular STING foci resulting from cGAMP or PC7A treatment follow a similar course of translocation from the endoplasmic reticulum (ER) to the ER–Golgi intermediate compartments (ERGIC) and the Golgi apparatus (Fig. 1e and Supplementary Fig. 2a). During transportation, STING forms clusters and phosphorylates TANK-binding kinase 1 (TBK1) and interferon regulatory factor 3 (IRF3; Fig. 1f), which initiates the downstream production of IFN-I proteins. In the presence of brefeldin A (BFA), which blocks protein trafficking between ER and Golgi^36^, both cGAMP and PC7A fail to trigger the production of phosphorylated TBK1 and IRF3 (p-TBK1/p-IRF3) production and proinflammatory cytokine expression (Fig. 1f and Supplementary Fig. 2b–d).

### PC7A binds to STING and forms biomolecular condensates

**Fig. 2:**
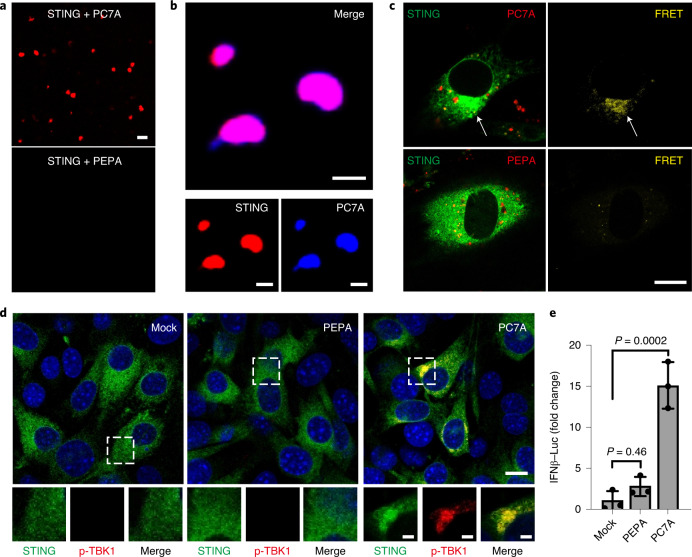
PC7A polymer induces STING condensation and immune activation. **a**, PC7A, but not PEPA, induces STING (Cy5-labelled) phase condensation after 4 h incubation. Scale bar, 10 μm. **b**, STING (4 μM, Cy5-labelled) and PC7A polymer (2 μM, aminomethylcoumarin acetate-labelled) are colocalized within the condensates. Scale bars, 5 μm. **c**, Hetero-FRET from GFP–STING to TMR–PC7A shows the colocalization of STING and PC7A in MEFs. Energy transfer was not observed from GFP–STING to TMR–PEPA. Cell culture conditions were identical to those described in Fig. 1. GFP (*λ*_ex_/*λ*_em_ = 488 nm/515 nm) and TMR (*λ*_ex_/*λ*_em_ = 555 nm/580 nm) signals are shown on the left in green and red, respectively. FRET signals (*λ*_ex_/*λ*_em_ = 488 nm/580 nm) are shown in yellow on the right. Scale bar, 10 μm. **d**, p-TBK1 is recruited into the STING–PC7A condensates. Scale bar, 10 μm. Insets: magnified images of the areas indicated by the white boxes. Scale bars, 2 μm. **e**, PC7A, but not PEPA, induces the expression of IFNβ–luciferase in ISG-THP1 cells. Data are mean ± s.d. *n* = 3 biologically independent experiments. Statistical analysis was performed using one-way analysis of variance (ANOVA). The confocal images in **a**–**d** are representative of at least three biologically independent experiments.

To investigate the biophysical mechanism of PC7A-mediated STING clustering and activation, we first determined the binding affinity between PC7A and STING (in human, amino acids 137–379, C-terminal domain) using isothermal titration calorimetry (ITC). STING binds strongly to PC7A (apparent dissociation constant (*K*_d_)= 72 nM) but weakly to other polymers with the same backbone, such as PEPA (apparent *K*_d_ = 671 nM; Supplementary Fig. 3a–c). Notably, polymers with cyclic side chains exhibit higher affinity to STING than linear analogues, and the seven-membered-ring of PC7A elicits the strongest binding. To investigate whether PC7A was sufficient to induce clustering of STING in vitro, we incubated cyanine-5 (Cy5)-labelled STING C-terminal domain (CTD) dimer with PC7A or PEPA at pH 6.5 (both P7CA and PEPA have apparent p*K*a values at 6.9, and remain as cationic unimers at pH 6.5). PEPA was used as a negative control due to its poor binding affinity to STING. After mixing of Cy5–STING and PC7A, liquid droplets were observed within minutes and grew over time; approximately 85% of STING proteins were present in the condensates after 4 h (Fig. 2a). Incubation of Cy5–STING with PC7A labelled with aminomethylcoumarin acetate confirmed colocalization of PC7A with STING puncta (Fig. 2b). Similar condensates were also observed in GFP–STING-expressing cell lysates after PC7A incubation (Supplementary Fig. 3d). The biomolecular condensates are hydrophobic as indicated by the increased fluorescence intensity and red-shifted maximum emission wavelength in a Nile-Red assay^37^ (Supplementary Fig. 3e). Fluorescence resonance energy transfer (FRET) from GFP–STING to tetramethylrhodamine (TMR)–PC7A further confirmed the formation of a biomolecular condensate consisting of PC7A and STING in mouse embryonic fibroblasts (MEFs) overexpressing human STING (Fig. 2c). The downstream protein product p-TBK1 was also found in this macromolecular cluster (Fig. 2d). By contrast, no obvious STING condensation or activation was observed when PEPA was used in these studies (Fig. 2 and Supplementary Fig. 3d). At pH 7.4, few PC7A–STING condensates were formed (Supplementary Fig. 3f) due to micellization of PC7A polymers above its p*K*a (6.9) and PEG shielding^38,39^.

### PC7A induces STING activation through polyvalent interactions

**Fig. 3:**
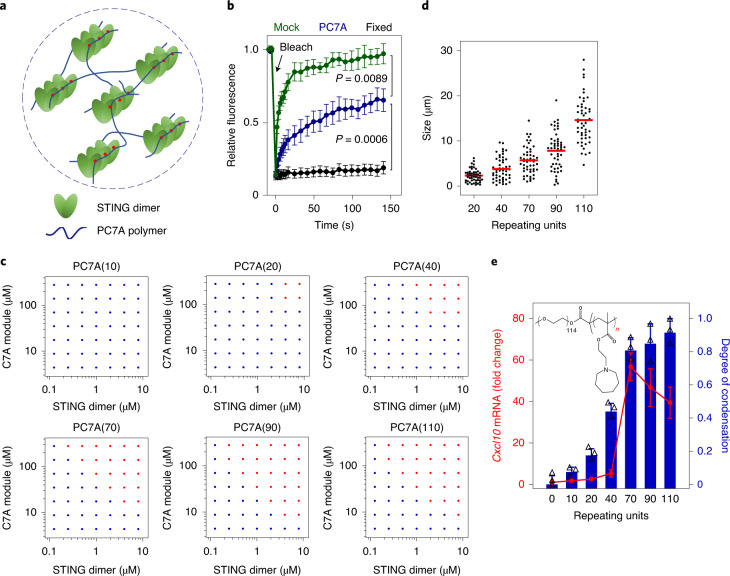
PC7A polymer induces STING condensation and immune activation through polyvalent interactions. **a**, Schematic of STING oligomerization and condensation driven by PC7A through polyvalent interactions. **b**, PC7A decreases the molecular mobility of GFP–STING in the condensates compared with free GFP–STING in MEFs. Bleaching was performed 24 h after PC7A treatment, and recovery was monitored over 150 s. Untreated (mock) and fixed cells were used as mobile and stationary STING controls, respectively. Data are mean ± s.d. *n* = 5 cells examined over two independent experiments. Statistical analysis was performed using one-way ANOVA. **c**, Biomolecular condensation of STING and PC7A is dependent on PC7A valency. The red dots indicate phase separation, and the blue dots indicate no phase separation. **d**, Size distributions of STING condensates induced by an increase in PC7A with higher PC7A valency. Condensate size was calculated as the average of longest and shortest axes. *n* = 50 condensates examined over two independent experiments. The red lines represent the average. **e**, STING activation in THP1 cells is correlated with the PC7A valency, with optimal *Cxcl10* expression induced by PC7A(70). Data are mean ± s.d. *n* = 3 biologically independent experiments. For the experiments shown in **c**–**e**, polymers with different repeating units were used at the same C7A modular concentrations.

Recent studies revealed that STING oligomerization after cGAMP binding is responsible for the recruitment and activation of downstream TBK1 and IRF3 proteins^18–21^. We hypothesized that PC7A polymer can serve as a supramolecular scaffold and directly engage polyvalent interactions to multimerize STING molecules for activation (Fig. 3a). To test this idea, we first labelled STING proteins using a FRET pair (TMR and Cy5) and mixed the two differentially labelled proteins at a ratio of 1:1. After addition of PC7A, we observed strong energy transfer from TMR to Cy5 (Supplementary Fig. 4a), indicating close proximity of STING dimers after polyvalent binding to PC7A. Fluorescence recovery after photobleaching (FRAP) experiments^40,41^ on STING–PC7A condensates revealed that, although both PC7A polymer and STING protein are exchangeable with surrounding molecules, PC7A exhibited a slower recovery rate than STING (Fig. 3b and Supplementary Fig. 4b,c).

To examine the effects of binding valence, we synthesized a series of PC7A polymers with an increasing number of repeating units. PC7A(*n*) refers to a polymer with *n* repeating units of the C7A methacrylate monomer. We incubated PC7A of increasing lengths with STING dimer under a matrix of concentrations in vitro to generate a phase diagram, which shows a minimum requirement of 20 repeating units for condensation (Fig. 3c). No phase separation was observed for PC7A(10). A higher degree of PC7A polymerization resulted in larger condensates (Fig. 3d and Supplementary Fig. 5a–c). For PC7A(110), more than 90% of STING proteins were found in the condensates, compared with 17% when PC7A(20) was used (Supplementary Fig. 5d). PC7A with a higher degree of polymerization exhibited lower phase reversibility and slower recovery rate of STING after photobleaching (Supplementary Fig. 5e,f). To investigate the relationship between condensate formation and STING activation in live cells, we treated THP1 cells with PC7A of varying lengths, and compared the *Cxcl10* mRNA expression levels. Longer polymers induced higher *Cxcl10* expression, with peak levels observed at 70 repeating units of PC7A (Fig. 3e). Further elongation of chain length (for example, 110) led to reduced *Cxcl10* expression, probably owing to the weaker signalling capacity of oversized condensates with excessive cross-linking and poor molecular dynamics^41,42^.

### PC7A binds to a distinct surface site from the cGAMP-binding pocket

The STING–PC7A condensates are sensitive to high concentrations of salt or the presence of other proteins. Although STING–PC7A condensates were formed at a physiological concentration of NaCl (150 mM), no phase separation was observed when the salt concentration was raised to 600 mM (Supplementary Fig. 6a,b). When bovine serum albumin (BSA) was added, the condensates decreased in number and size (Supplementary Fig. 6c). To further investigate the specificity of PC7A-induced condensate, we labelled STING with Cy5 and BSA with boron-dipyrromethene (BODIPY) dyes. In the presence of PC7A, only Cy5–STING was present in the condensates, whereas the majority of BODIPY–BSA was excluded (Supplementary Fig. 6d). As controls, mixtures of BSA and PC7A, or STING and BSA did not form condensates.

**Fig. 4:**
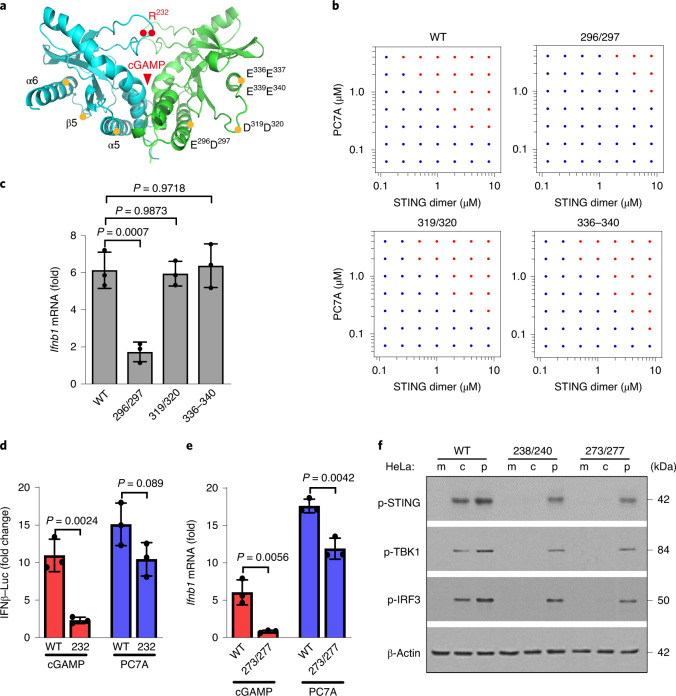
STING condensation and activation by PC7A polymer occurs through a distinct binding site from cGAMP. **a**, Schematic of site-directed mutagenesis on the STING structure. Mutation sites are distinct from the cGAMP-binding pocket. **b**,**c**, STING mutation of E296A/D297A abolishes STING condensation (**b**) and immune activation (**c**) in response to PC7A. Other mutations in STING do not affect PC7A-induced STING activation. Data are mean ± s.d. *n* = 3 biologically independent experiments. Statistical analysis was performed using one-way ANOVA. **d**–**f**, PC7A retains immune activity in several cGAMP-resistant STING variants. R232H in THP1 cells (**d**) or R238A/Y240A in HeLa cells (**f**) abrogates cGAMP binding. Q273A/A277Q (**e**,**f**), which disrupts the tetramer interface and cGAMP-mediated STING oligomerization, abolishes STING activation by cGAMP but not by PC7A. Data are mean ± s.d. *n* = 3 biologically independent experiments. Statistical analysis was performed using two-tailed Student’s *t*-tests. c, cGAMP; m, mock; p, PC7A polymer.

On the basis of the pH (Supplementary Fig. 3f) and salt (Supplementary Fig. 6a,b) effects on the PC7A–STING interactions and computational modelling (data not shown), we hypothesized that negatively charged surface sites on STING may be responsible for PC7A binding. To test this hypothesis, we constructed STING mutants with several negatively charged amino acids in the α5–β5–α6 region replaced by alanine and investigated their PC7A-binding affinity, phase condensation and STING activation both in vitro and in live cells. Notably, the mutation of two acidic residues (E296A/D297A) on the α5 helix was sufficient to abolish polymer binding and biomolecular condensation, whereas two other mutants (D319A/D320A and E336A/E337A/E339A/E340A) exhibited marginal effects (Fig. 4a,b and Supplementary Table 1). We next transfected HEK293T cells with mutant STING plasmids and measured downstream activation. Consistent with the abrogation of PC7A binding and condensation, the E296A/D297A mutant was deficient in forming condensate structures and inducing TBK1 phosphorylation and *Ifnb1*/*Cxcl10* expression in cells (Fig. 4c and Supplementary Fig. 7a,d). By contrast, these STING mutants did not impact cGAMP-mediated STING activation (Supplementary Fig. 7b,c). Together, these data suggest that the Glu 296-Asp 297 site on the α5 helix of STING, which is distinct from the cGAMP-binding site, is responsible for PC7A binding and induced activation.

Endogenous STING agonists (cGAMP or other CDNs) bind to the STING dimer interface covered by a lip of four-stranded antiparallel β-sheet (human amino acids 219–249)^43,44^. A natural STING variant (R232H) that occurs in ~14% of the human population exhibits a reduced response to small-molecule STING agonists^45^. As PC7A binds to a STING site that is different from the cGAMP-binding pocket, we tested the biological activity of PC7A in THP1 cells harbouring the STING R232H variant. Whereas the cGAMP response was abrogated in these cells as expected, PC7A still had the ability to elevate IFNβ–luciferase expression (Fig. 4d). Additional studies in mutant HeLa cells (R238A/Y240A or Q273A/A277Q mutations that abolish cGAMP binding or prevent STING oligomerization after cGAMP binding, respectively)^20,21^ show persistent PC7A-induced STING activation, whereas cGAMP-mediated effects were abolished (Fig. 4e,f and Supplementary Fig. 7e–h). Collectively, these results demonstrate that PC7A stimulates STING through cGAMP-independent mechanisms.

### PC7A prolongs innate activation in vivo and synergizes with cGAMP in antitumour immunity

In vitro cell culture studies show that PC7A NPs generated more-durable STING activation compared with free cGAMP (Fig. 1a–c). To test whether PC7A NPs prolong STING activation in vivo, we intratumourally injected cGAMP (50 μg), PC7A NPs (50 μg) and cGAMP-loaded PC7A NPs (2.5 μg/50 μg) into MC38 tumours (~100 mm^3^) and measured the expression of interferon-stimulated genes in both tumours and the draining lymph nodes over time. Owing to the ability of PC7A for STING activation and cytosolic delivery of cGAMP, we chose a lower dose of cGAMP (~5 wt% loading) in cGAMP–PC7A NPs for the majority of in vivo studies. cGAMP–PC7A NPs were prepared using a base titration method, resulting in spherical micelles of 29.9 ± 2.5 nm (mean ± s.d.) in diameter and over 90% loading efficiency (Supplementary Fig. 8). Consistent with our in vitro studies, mice treated with free cGAMP showed rapid *Ifnb1*/*Cxcl10* expression 6 h after intratumoural injection, while the activity decreased considerably over 48 h in both tumour and nodal tissues (Supplementary Fig. 9). By contrast, PC7A-induced STING activity was minimal at 6 h but reached the maximum level at 24 h. cGAMP–PC7A NPs yield the most optimal STING activity profile, which exhibited a rapid increase in *Ifnb1*/*Cxcl10* expression compared with PC7A (50 μg) at 6 h and, in contrast to free cGAMP, this response was also sustained over 48 h.

**Fig. 5:**
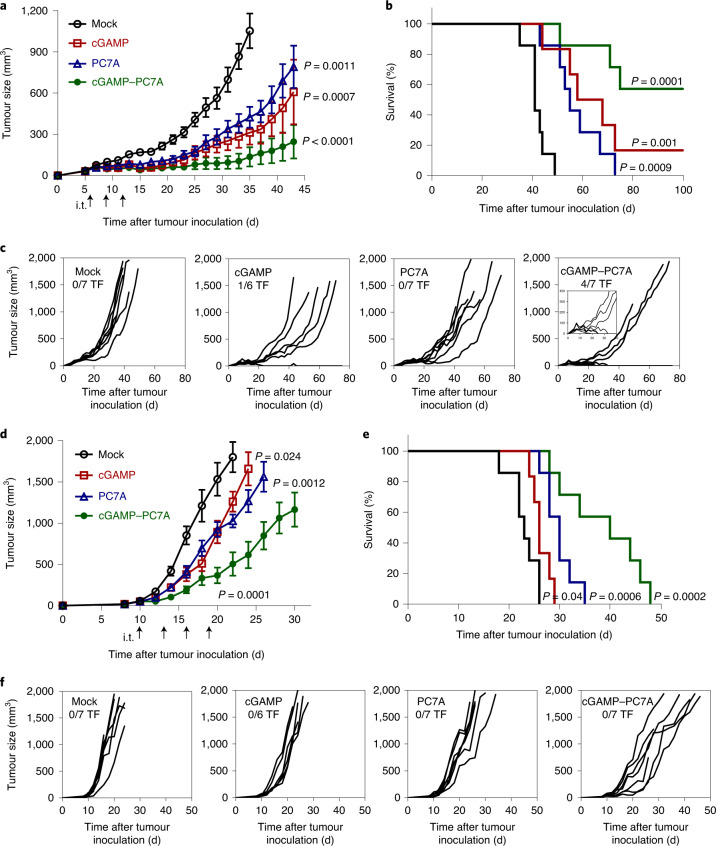
PC7A and cGAMP show synergistic antitumour efficacy in tumour-bearing mice. **a**–**f**, MC38 (**a**–**c**) and TC-1 (**d**–**f**) tumour-bearing mice were injected intratumourally (i.t.; arrows) with 5% glucose (mock), cGAMP (2.5 μg), PC7A NPs (50 μg) or cGAMP-loaded PC7A NPs at the indicated time points. Mean tumour volume (**a**,**d**), Kaplan–Meier survival curves (**b**,**e**) and spider plots of individual tumour growth curves (**c**,**f**) are shown. PC7A NPs or cGAMP alone offer some degree of immune protection. cGAMP-loaded PC7A NPs confer a synergistic antitumour immune response, with significantly improved survival; 4 out of 7 mice in the MC38 model were tumour-free (TF). In the tumour growth studies (**a** and **d**), data are mean ± s.e.m. *n* = 7 (mock), *n* = 6 (cGAMP), *n* = 7 (PC7A) and *n* = 7 (cGAMP–PC7A) biologically independent mice in each tumour model. For **a** and **d**, statistical analysis was performed using two-tailed Student’s *t*-tests (versus mock). For **b** and **e**, statistical analysis was performed using Mantel–Cox tests.

Next, we investigated the antitumour efficacy in MC38 and TC-1 tumour models (Fig. 5). In MC38 tumours, we performed three intratumoural injections of free cGAMP (2.5 μg or 50 μg; high-dose data are provided in Supplementary Fig. 10a), PC7A NPs (50 μg) or cGAMP–PC7A NPs (2.5 μg or 50 μg) when tumours reached ~50 mm^3^ in size. As a negative control, we injected mice with a 5% glucose solution (all of the treatment groups were prepared in 5% glucose solutions). The results show that all of the mice in the control group died within 50 d after MC38 inoculation. Groups that were treated with cGAMP (2.5 μg) or PC7A alone showed notably extended the survival compared with the control group, while the difference between the two treatment groups was not statistically significant. cGAMP–PC7A NP treatment achieved the most efficacious outcome, with 4 out of 7 mice remaining tumour free over 100 d after tumour inoculation. In the TC-1 model, all of the mice in the control group died within 26 d. cGAMP or PC7A alone conferred a minor degree of immune protection, extending the median survival time by 4 d or 8 d, respectively. The cGAMP–PC7A NP treatment showed significantly improved tumour growth inhibition and long-term survival compared with either treatment alone.

In MC38 tumours, a high dose (50 μg) of free cGAMP treatment did not lead to significantly improved tumour growth inhibition compared with the low-dose group (2.5 μg; Supplementary Fig. 10a). By contrast, systemic side effects were observed at the higher cGAMP dose, as evidenced by the elevated levels of alanine transaminase and aspartate transaminase (liver), urea (kidney) and systemic cytokine (for example, IL-10; Supplementary Fig. 10b–e). cGAMP–PC7A NP treatment did not show a significant increase in toxic side effects compared with the control group.

Previous studies have shown an association between elevated IFN-I production and increased tumour infiltration of PD-1^+^ cytotoxic T lymphocytes^7,46–48^. We hypothesized that STING activation by cGAMP–PC7A NPs may synergize with PD-1 blockade. We found that the combination provided significantly improved efficacy—100% of the mice remained tumour-free after 100 d in the mouse MC38 colorectal tumour model (Supplementary Fig. 11a–c). The therapeutic efficacy was also improved in the more aggressive TC-1 tumour model; more than 50% of the mice bearing TC-1 tumours survived for longer than 45 d (Supplementary Fig. 11d–f).

### STING status and immune cell type on PC7A-induced antitumour immunity

Using an in vivo cell killing assay, our previous study showed that the generation of antigen-specific T cells by the PC7A NP vaccine was dependent on the STING–IFN-I pathway^17^. To confirm the importance of the STING pathway and to determine whether host or cancer cell STING status has a more dominant role in PC7A-induced antitumour immunity, we performed a tumour growth inhibition assay in host *Tmem173*^−/−^ (which encodes STING) mice + wild-type (WT) MC38 tumours and WT mice + *Tmem173*^−/−^ MC38 tumours (Supplementary Fig. 12a–c). Without treatment, WT MC38 cancer cells grew faster in *Tmem173*^−/−^ mice than in WT mice, indicating the role of the STING pathway in immune protection by the host alone. The antitumour efficacy improvement of PC7A and cGAMP–PC7A NPs was abolished in *Tmem173*^−/−^ animals compared with the WT mice. By contrast, comparable antitumour efficacy by PC7A and cGAMP–PC7A NPs was observed when treating WT mice with *Tmem173*^−/−^ tumours versus WT MC38 tumours.

To further investigate the immune-cell-dependent contribution to antitumour immunity, we evaluated tumour growth inhibition by antibody blockade of CD8 T cells, NK cells and in CD11c-DTR transgenic mice with depletion of DCs^49^. Blockade of CD8 T cells abolished the antitumour efficacy of PC7A treatment whereas blockade of NK cells showed minimal effect (Supplementary Fig. 12d,e). Results in CD11c-DTR mice showed that DC depletion reduced the therapeutic efficacy after treatments, albeit to a lesser extent when compared with CD8 T-cell blockade (Supplementary Fig. 12f).

### STING activation in human tissues

**Fig. 6:**
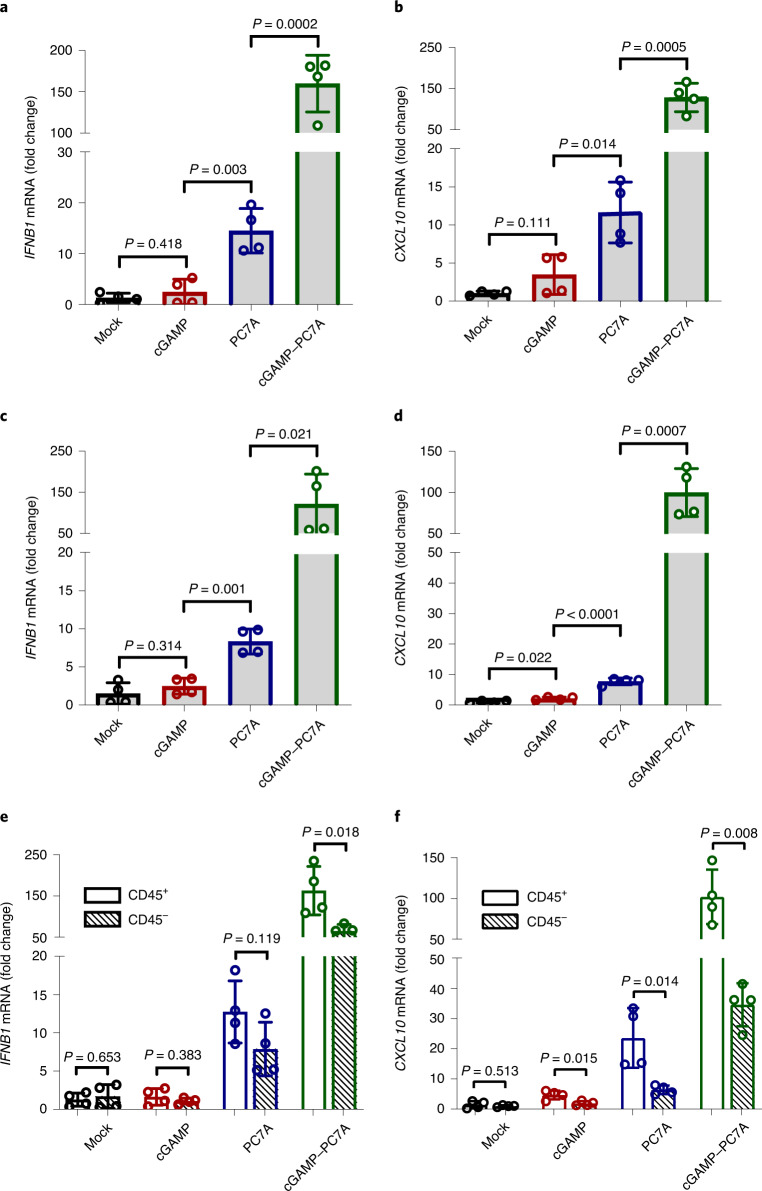
PC7A and cGAMP show synergistic STING activation in fresh human tissues. **a**–**f**, Free cGAMP alone is unable to activate STING, whereas PC7A NPs and cGAMP-loaded PC7A NPs demonstrate effective STING activation. Fresh surgically resected sentinel lymph nodes (SLN) (**a**,**b**) or squamous cell carcinoma samples from the base of tongue (SCC-BOT) (**c**–**f**) were divided into multiple sections (1–5 mm^3^) and injected with 5% glucose, free cGAMP, PC7A NPs or cGAMP-loaded PC7A NPs in 5% glucose solutions. *IFNB1* (**a**,**c**,**e**) and *CXCL10* (**b**,**d**,**f**) gene expression was measured after 24 h incubation. **e**,**f**, The CD45^+^ cell population exhibited an enhanced level of STING activation compared with CD45^−^ cells. Data are mean ± s.d. *n* = 4 SLN sections from the same patient in **a** and **b**. *n* = 4 SCC-BOT tumour sections from the same patient in **c**–**f**. Statistical analysis was performed using two-tailed Student’s *t*-tests.

To examine the translational potential, we investigated the feasibility of STING activation in human tissues. We acquired freshly resected squamous cell carcinoma from the base/lateral of tongue, cervical tumour tissues and a sentinel lymph node. We locally injected these tissues with cGAMP, PC7A NPs or cGAMP–PC7A NPs, incubated them in cell culture medium for 24 h at 37 °C, and detected IFN-related gene expression. Free cGAMP had a marginal effect on *IFNB1* and *CXCL10* mRNA expression over the control due to limited bioavailability. By contrast, PC7A NPs elevated downstream signals by fivefold to twentyfold. A substantial increase in cytokine expression (100–200-fold; Fig. 6a–d and Supplementary Fig. 13) was observed after treatment with cGAMP–PC7A NPs in all of the tissue types. Notably, after treatment with PC7A NPs and cGAMP–PC7A NPs, CD45^+^ myeloid cell populations in the tumour showed a higher level of STING activation compared with CD45^−^ cells (Fig. 6e,f), indicating that leukocytes, rather than cancer cells, are the primary targets for STING-mediated immunomodulation by NPs.

## Discussion

The therapeutic strategies described in this study take advantage of non-canonical STING activation by a synthetic polymer with cell intrinsic pathways by cGAMP for cancer immunotherapy. First, we determined a distinctive surface-binding site on the STING protein by the PC7A polymer that is different from the binding pocket of cGAMP or other CDNs. Although a previous report showed that the PC7A NP vaccine worked through STING for T-cell activation^17^, it is not clear whether PC7A competes with cGAMP for the same binding site at the STING dimer interface. The discovery of non-competitive binding sites formulates a basis to combine PC7A with cGAMP for synergistic STING activation while enabling PC7A to activate cGAMP-resistant STING variants (Fig. 4d–f). In humans, STING consists of several haplotypes (for example, 14% of the human population have the R232H phenotype) that exhibit reduced innate activity in response to CDN agonists^50,51^. PC7A presents an alternative STING activation strategy in these STING-variant patient populations. Second, we uncovered a PC7A-induced protein condensation mechanism for STING activation. We used a synthetic polymer to induce polyvalent phase condensation for biological activation. Phase condensates are shown to impact a broad range of biological processes and are under intensive investigations in biophysics and cell biology^29,30^. Here we provide a proof of concept to install polymer-induced protein condensation as an emerging bioengineering principle for biological interrogation and pharmaceutical development.

STING remains a promising target for cancer immunotherapy, but several small-molecule STING agonists showed limited efficacy and dose-limiting toxicity in early-stage clinical trials^27,28^. Here, intratumoural injection of a high dose of cGAMP (50 μg) did not lead to significant tumour growth inhibition compared with a low dose (2.5 μg), but resulted in increased systemic toxicity (Supplementary Fig. 10), corroborating clinical observations. We attribute the limited therapeutic window to the poor pharmacokinetics and mechanism of action in STING activation. Owing to its small size (674 Da) and water solubility, blood perfusion can quickly remove cGAMP from the tumour site to systemic circulation, limiting STING activation to a few hours inside tumours (Supplementary Fig. 9).

Compared with cGAMP, the PC7A polymer induces a slower but more sustained STING activity in vitro and in vivo. We attribute this kinetic difference to several factors. First, endosomal escape followed by cytosolic transport to reach ER-bound STING target is probably faster for cGAMP than the PC7A polymer (molecular mass (MM) = 21 kDa). Second, cGAMP-induced conformational change of STING and subsequent oligomerization^20,21^ may also occur faster than PC7A-induced STING condensate formation for immune activation. Finally, buffering of endosomal pH and disruption of endosomal membranes by PC7A deter STING degradation through the endosome–lysosome pathway. With the ability of PC7A to activate STING and cytosolic delivery of cGAMP, we demonstrate that cGAMP–PC7A NPs achieved rapid and sustained STING activation across 6–48 h in both MC38 tumours and draining lymph nodes (Supplementary Fig. 9), which enable an optimal time window for DC maturation and T-cell priming (normally requires 1–2 d)^52,53^. This is supported by the synergistic therapeutic outcomes of cGAMP–PC7A NPs in MC38 and TC-1 tumour treatment over single therapy alone.

A growing number of studies report the importance of STING pathway in cancer immunotherapy^12–17^. However, it is unclear whether STING activity in the cancer cells, immune cells or stromal cells have a more critical role in antitumour immunity. Our studies revealed the importance of host STING activity in cGAMP–PC7A NP therapy (Supplementary Fig. 12). Data also show that tumour growth inhibition is abolished by antibody blockade of CD8 T cells but not NK cells, indicating that CD8 T cells are the ultimate effector cells against tumours. We also show partial reduction of antitumour efficacy in DC-depleted mice, suggesting that additional immune cells (such as macrophages and B cells) or stromal cells (such as fibroblasts) may also contribute to the T-cell-mediated antitumour immunity. Further investigations are warranted to elucidate the contributions from other immune cell types or subset of immune cells (for example, tumour-resident CD103^+^ DCs)^11,54^, which may help to identify key biomarkers for clinical translation.

In summary, this study highlights the use of a synthetic polymer to induce STING condensation for activation of an important innate immune pathway with spatiotemporal dynamics distinct from a natural STING ligand. Combination of polyvalent STING activation by PC7A with cell-intrinsic cGAMP stimulation further offers a synergistic and robust strategy to mount antitumour immunity for cancer immunotherapy.

## Methods

### Synthesis of polymers

Monomers including 2-hexamethyleneiminoethyl methacrylate (C7A-MA), 2-(4-methylpiperidineleneimino)ethyl methacrylate (C6S1A-MA), 2-heptamethyleneiminoethyl methacrylate (C8A-MA), 2-diisopropylaminoethyl methacrylate (DPA-MA) and 2-ethylpropylaminoethyl methacrylate (EPA-MA) were synthesized according to previous publications^39,55^. PEG-*b*-PR copolymers were synthesized using an atom transfer radical polymerization method. Poly(ethylene glycol)-*b*-poly(2-hexamethyleneiminoethyl methacrylate) with 70 repeating units, that is, PC7A(70), is used as an example to illustrate the procedure. First, C7A-MA (1.5 g, 7 mmol), MeO-PEG_114_-Br (0.5 g, 0.1 mmol, Sigma-Aldrich) and N,N,N′,N′′,N′′-pentamethyldiethylenetriamine (PMDETA, 21 μl, 0.1 mmol, Sigma-Aldrich) were dissolved in a mixture of 2-propanol (2 ml) and dimethylformamide (2 ml) in a Schlenk flask. Oxygen was removed by three cycles of freeze–pump–thaw, then CuBr (14 mg, 0.1 mmol, Alfa Aesar) was added under nitrogen protection. Polymerization was performed in vacuo at 40 °C overnight. After polymerization, the reaction mixture was diluted in tetrahydrofuran (10 ml), and then passed through a neutral Al_2_O_3_ column to remove the catalyst. The organic solvent was removed by rotary evaporation. The residue was dialysed in distilled water and lyophilized to obtain a white powder. After syntheses, the product was characterized using ^1^H NMR and gel permeation chromatography. The four other polymers, including PC6S1A, PC8A, PDPA and PEPA, were all synthesized with 70 repeating units. PC7A polymers with different repeating units were synthesized by adjusting the initial ratio of C7A-MA monomer over the MeO-PEG_114_-Br initiator.

The synthesis of dye-conjugated copolymers was performed according to a similar procedure^39,55^. Primary amino groups (aminoethyl methacrylate or AMA-MA, Polysciences) were introduced into each polymer chain by controlling the feeding ratio of AMA-MA monomer to the initiator (3:1). After synthesis, PEG-*b*-(PR-*r*-AMA) was dissolved in dimethylformamide, and dye-*N*-hydroxylsuccinimidal ester was added (3 molar equivalences to the primary amino group, Lumiprobe). After overnight reaction, the copolymer was purified using ultracentrifugation (MM = 10 kDa cut-off) three times to remove free dye molecules. The product was lyophilized and stored at −80 °C.

### Preparation of micelle NPs

Micelle NPs for cellular studies were prepared according to a solvent evaporation method as previously reported^39,55^. In brief, polymer (4 mg) was first dissolved in methanol (0.4 ml) and then added dropwise into distilled water (3.6 ml) under sonication. Methanol was removed by ultrafiltration (MW = 100 kDa cut-off) three times with fresh distilled water. Sterile PBS was added to adjust the concentration to 200 μM as a stock solution.

cGAMP-loaded NPs were prepared by mixing 2′3′-cGAMP in PC7A polymer solution containing 5% d-glucose at pH 4, and then adjusted to pH 7.4 using NaOH. After micelle formation, the NPs were analysed using dynamic light scattering to measure size and zeta potential, and transmission electron microscopy to analyse particle morphology. The cGAMP loading efficiency (>90%) was quantified using high-performance liquid chromatography.

### Expression, purification and labelling of recombinant STING proteins

A human STING CTD (amino acid sequence 139–379) plasmid containing a His_6_ tag encoded in the pET-SUMO vector (provided by Z. J. Chen, UT Southwestern) was used as a template to generate the E296A/D297A, D319A/D320A and E336A/E337A/E339A/E340A mutants using a Q5 site-directed mutagenesis kit (NEB). Overexpression of the WT or mutant protein was induced in *Escherichia coli* strain BL21/pLys with 0.8 mM isopropyl-β-d-thiogalactoside at 16 °C for 18 h. Bacterial cells were collected, suspended (50 mM Tris-Cl, 300 mM NaCl, 20 mM imidazole, pH 8.0) and disrupted by sonication on ice. Cellular debris was removed by centrifugation at 20,000*g* at 4 °C for 1 h. The supernatant was loaded onto a Ni^2+^-nitrilotriacetate affinity resin (Qiagen). After 4 h incubation at 4 °C, the resin was rinsed three times with washing buffer (50 mM Tris-Cl, 1 M NaCl, 20 mM imidazole, pH 8.0). The SUMO tag was then removed by digesting the proteins using ULP1 SUMO protease at 4 °C overnight. Proteins were eluted with elution buffer (20 mM Tris-Cl, 50 mM NaCl, 20 mM imidazole, pH 7.5). Subsequently, the eluted proteins were analysed by size-exclusion chromatography using a Superdex 200 column (GE Healthcare), and the fractions were collected, concentrated and dialysed against a buffer containing 25 mM HEPES and 150 mM NaCl (pH 7.5)^43^.

For dye conjugation, the protein solution was mixed with Cy5-NHS in NaHCO_3_ (pH 8.4) at 4 °C overnight. Free dye molecules were removed using a desalting column (7 K, Thermo Fisher Scientific). Dye-labelled proteins were collected, concentrated and used in phase-separation studies.

### ITC analysis

A MicroCal VP-ITC was used to measure the binding affinity between protein and polymer. STING dimer concentration was held at 12.5 μM and PC7A(70) at 10 μM. The titrations were performed at 20 °C in a buffer containing 25 mM HEPES and 150 mM NaCl (pH 6.5). Twenty-nine injections were performed in 3 min spacing time. The titration traces were integrated using NITPIC v.1.2.7, the curves were fitted using SEDFHAT v.15.2b and the figures were prepared using GUSSI v.1.4.2.

### Nile Red assay

The Nile Red assay is used to study protein–protein interactions and interruptions in protein structure^37^. In brief, Nile Red (final concentration 5 μM, Thermo Fisher Scientific), STING dimer (2.1 μM) and PC7A (0 μM, 0.6 μM, 1.2 μM, 3 μM, 6 μM or 12 μM) were mixed for 4 h. Their maximum excitation wavelengths and fluorescence intensities were recorded on a fluorescence spectrophotometer (Hitachi F-7000 model).

### Phase condensation assay

WT or mutant human STING CTD (Cy5-labelled) was mixed with PC7A polymers of varying repeating units in a 96-well glass plate (coated with mPEG-silane) at 25 °C. After 4 h, the mixture was centrifuged at 13,000*g* for 5 min, and the supernatant was transferred to another plate. Fluorescence intensity of the supernatant was measured using a plate reader (CLARIOstar). Data are representative of at least three independent measurements. The degree of condensation (*D*) was calculated using the following equation:

$$D_i = \frac{{F_0 - F_i}}{{F_0}}$$

where *F*_*i*_ is the fluorescence intensity of the supernatant for a specific group *i*, and *F*_0_ is the Cy5–STING intensity at the same concentration without PC7A addition.

For phase reversibility assays, STING CTD (Cy5-labelled) and PC7A polymer were first mixed. After condensate formation, the mixture was diluted ten times in pH 6.5 HEPES buffer, and shaken on a plate shaker for 24 h. The fluorescence intensity of the supernatant was measured, and reversibility (*R*) was calculated using following equation:

$$R_i = \frac{{D_{i - }D_{R_{i}}}}{{D_i}}$$

where $D_{R_{i}}$ was the new *D* value after 24 h recovery.

For microscopy examination, STING protein (Cy5-labelled) was mixed with PC7A polymer in a four-well glass chamber (Thermo Fisher Scientific; coated with mPEG-silane) at 25 °C, and images were acquired over a 140 s time course at intervals of 4 s using the built-in software (ZEN v.2.6) of the Zeiss 700 confocal laser scanning microscope. Size was calculated as the average of the longest and shortest axis of each condensate. The size distribution was plotted using GraphPad Prism 7.

### Animals and cells

All of the animals were maintained at the animal facilities under specific-pathogen-free conditions and all animal procedures were performed with ethical compliance and approval by the Institutional Animal Care and Use Committee at the University of Texas Southwestern Medical Center. Female C57BL/6 mice (aged 6–8 weeks) were obtained from the UT Southwestern breeding core. Host *Tmem173*^−*/*−^ C57BL/6 mice^56^ were provided by Y.-X. Fu and CD11c-DTR transgenic C57BL/6 mice were purchased from the Jackson Laboratory. Mice were housed in a barrier facility under a 12 h–12 h light–dark cycle and maintained on standard chow (2916, Teklad Global). The temperature range for the housing room is 68–79 °F (average is around 72 °F) and the humidity range is 30–50% (average is around 50%).

GFP–STING MEFs (provided by N. Yan, UT Southwestern), and HEK293T (ATCC), B16F10 (ATCC), MC38 (ATCC), *Tmem173-KO* MC38 (provided by Y-X. Fu)^56^, TC-1 (provided by T. C. Wu, John Hopkins University) cells were cultured in complete DMEM medium supplemented with 10% fetal bovine serum (FBS). THP1 cells (ATCC) were cultured in RPMI medium supplemented with 10% FBS and 0.05 mM β-mercaptoethanol (β-ME). All cells were grown at 37 °C in 5% CO_2_. THP1 monocytes were differentiated into macrophages by phorbol 12-myristate 13-acetate (PMA, 150 nM, InvivoGen) before use.

In the cell mutagenesis assay, GFP-tagged full-length WT STING plasmid (provided by N. Yan) was used as a template to generate E296A/D297A, D319A/D320A and E336A/E337A/E339A/E340A mutants. HEK293T cells were transfected with lipofectamine 2000 (Invitrogen) carrying full-length WT or mutant STING-GFP plasmid for 24 h and allowed to recover for 12 h before use. WT or R232H THP1 reporter cells were purchased from Invitrogen. R238A/Y240A and single or dual Q273A/A277Q HeLa mutants (provided by Z. J. Chen)^20,21^ were used as cGAMP-resistant STING mutant cells.

### Microscopy

Cells were grown in a four-well glass chamber and treated with cGAMP or PC7A polymer for the indicated time. In the STING degradation assay, LysoTracker Red DND-99 (Thermo Fisher Scientific) was used to stain lysosomes in live cells. In the STING trafficking assay, cells were fixed in 4% paraformaldehyde, then permeabilized and stained for ER (Calnexin, 1:200, Abcam), ERGIC (p58, 1:1,000, Sigma-Aldrich), Golgi (GM130, 1:50, BD Biosciences) or p-TBK1 (Ser 172, 1:50, Cell Signaling Technologies) using an immunofluorescence kit (Cell Signaling Technologies). Samples were mounted in prolong gold antifade with DAPI stain (Thermo Fisher Scientific) and imaged using the built-in software (ZEN 2.6) of the Zeiss 700 confocal laser scanning microscope with a ×63 oil-immersion objective. ImageJ v.1.52d was used to quantify colocalization using the Pearson’s correlation coefficient. Data are representative of at least 20 cells. In the inhibitor assay, cells were pretreated with BFA (10 μM, Selleckchem) for 1 h before cGAMP/PC7A addition.

### FRAP experiments

The FRAP method is a versatile tool for determining the diffusion and exchange properties of biomacromolecules^57^. Both in vitro and cellular FRAP experiments were performed using a Zeiss 700 confocal laser scanning microscope at 25 °C. In a typical procedure, a 2-μm-diameter spot in the condensation was photobleached with 100% laser power for 5 s using a 633 nm laser. Images were acquired over a 150 s time course at intervals of 4 s. Fluorescence intensity of the region of interest was corrected by an unbleached control region and then normalized to the prebleached intensity of the region of interest. At least five biologically independent samples were measured. The mean intensity of the bleached spot was fit to a single exponential model^32^ using Graph Pad Prism 7 software.

### Western blot analysis

All solutions were purchased from Bio-Rad and antibodies against STING (1:1,000), p-STING (S366, 1:1,000), p-TBK1 (Ser 172, 1:1,000) and p-IRF3 (Ser 369, 1:1,000) were obtained from Cell Signaling Technologies. In brief, cells were lysed in SDS sample buffer (with protease and phosphatase inhibitor cocktail) and heated for denaturation. Supernatant was loaded onto a 4–15% Mini-PROTEAN gel (Bio-Rad), and run at 50 V for 20 min followed by 100 V for 60 min. Electrotransfer was performed using 100 V for 60 min on ice. After transfer, the membrane was blocked either in 5% non-fat milk or BSA (phosphorylated protein) for 1 h at room temperature, and incubated with primary antibodies overnight at 4 °C. Goat anti-mouse or goat anti-rabbit IgG HRP-linked secondary antibody (1:3,000, Bio-Rad) was used for 1 h at room temperature before detection on X-ray film (GE Healthcare). The membrane was stripped in stripping buffer for 30 min and reused for β-actin (Sigma-Aldrich) detection.

### RT–qPCR

Total RNAs were extracted from cells or human tissues using the RNeasy mini kit (Qiagen). RNA quantity and quality were confirmed using the NanoDrop (DeNovix DS-11) system. Genomic DNA was removed and cDNA was synthesized using an iScript gDNA clear cDNA synthesis kit (Bio-Rad). Bio-Rad SsoAdvanced universal SYBR green supermix and CFX connect real-time system were used for PCR analysis. Results were corrected by *ACTB* or *GAPDH* in Excel Office 365 and plotted in Graph Pad Prism 7. The DNA primers used were as follows: mouse *Ifnb1*: ATGAGTGGTGGTTGCAGGC, TGACCTTTCAAATGCAGTAGATTCA; mouse *Cxcl10*: GGAGTGAAGCCACGCACAC, ATGGAGAGAGGCTCTCTGCTGT; mouse *Actb*: ACACCCGCCACCAGTTCGC, ATGGGGTACTTCAGGGTCAGGATA; human *IFNB1*: GTCTCCTCCAAATTGCTCTC, ACAGGAGCTTCTGACACTGA; human *CXCL10*: TGGCATTCAAGGAGTACCTC, TTGTAGCAATGATCTCAACACG; human *ACTB*: GGACTTCGAGCAAGAGATGG, AGGAAGGAAGGCTGGAAGAG; human *GAPDH*: ATGACATCAAGAAGGTGGTG, CATACCAGGAAATGAGCTTG.

### Evaluation of STING activation in tumour-bearing mice

Mice were subcutaneously inoculated with MC38 cells (1 × 10^6^) into the right flank. One intratumoural injection of different agents (50 μl of 5% glucose, 50 μg PC7A polymer, 2.5 or 50 μg cGAMP or a formulation with 2.5 μg cGAMP in 50 μg PC7A NPs) was performed when the tumour size reached 100 ± 20 mm^3^. Mice were euthanized at different time points after injection, and tumours and draining lymph nodes were collected. Total RNAs were extracted by TRIzol (Invitrogen), and the expression of interferon-stimulated genes (*Ifnb1* and *Cxcl10*) was measured using quantitative PCR with reverse transcription (RT–qPCR).

### Safety studies

Mice were subcutaneously inoculated with MC38 cells (1 × 10^6^) into the right flank. Intratumoural injections of different agents (50 μl of 5% glucose, 50 μg PC7A polymer, 2.5 or 50 μg cGAMP or a formulation with 2.5 μg cGAMP in 50 μg PC7A NPs) were performed when the tumour size reached ~50 mm^3^ (around day 6). Two additional injections were performed on days 9 and 12. One day after the final administration, 1 ml of blood sample was collected from each mouse without heparinization and then centrifuged at 4,000 r.p.m. for 5 min to obtain serum. The activities of alanine aminotransferase (ALT), aspartate aminotransferase (AST) and urea were measured using specific kits (Abcam, 105134, 105135, 83362, respectively). The systemic concentration of interleukin-10 was measured using an enzyme-linked immunosorbent assay (Invitrogen, 88-7105-22). Statistical analysis was performed using GraphPad Prism 7.

### Tumour therapy experiments

Mice were subcutaneously inoculated with MC38 cells (1 × 10^6^) or TC-1 cells (1 × 10^5^) into the right flank. Tumour size was measured every 2 d or 3 d using digital callipers, and tumour volume was calculated as 0.5 × length × width^2^. On reaching sizes of ~50 mm^3^, tumours were injected with different STING agonists (50 μl of 5% glucose, 50 μg PC7A polymer, 2.5 μg or 50 μg cGAMP, or 2.5 μg cGAMP in 50 μg PC7A NPs), and some of the groups were intraperitoneally injected with 200 µg depletion antibodies (anti-mCD8α, BioXcell, BP0117 or anti-mNK1.1, BioXcell, BP0036) or 200 µg checkpoint inhibitors (anti-mPD-1, BioXcell, BE0146) every 3 d for comparison or synergy evaluation. For systemic DC depletion, CD11c-DTR transgenic mice were injected intraperitoneally with 100 ng diphtheria toxin (Sigma-Aldrich) every 3 d after tumour inoculation. Mice were injected three times in the MC38 model and four times in the TC-1 model with STING agonist treatments spaced 3 d apart. Mice were euthanized at a tumour burden endpoint of 2,000 mm^3^. Statistical analysis was performed using GraphPad Prism 7.

### Evaluation of STING activation in resected human tissues

Patients provided consent for the use of biospecimens for research as approved by the UT Southwestern Institutional Review Board. Freshly resected human tissues (squamous cell carcinoma from the base/lateral of tongue, cervical tumour tissues and a sentinel lymph node) were rinsed and divided into several sections (1–5 mm^3^) using a scalpel, followed by injection at multiple sites using 5% glucose control, free cGAMP (80 ng), PC7A polymer (50 μg) or cGAMP–PC7A NPs (80 ng cGAMP in 50 μg PC7A NPs) in 5% glucose solution within 30 min of resection. Each section was cultured in 0.5 ml RPMI 1640 medium (supplemented with 10% heat-inactivated human serum, 1% insulin-transferrin-selenium, 1% GlutaMAX, and 1% penicillin–streptomycin) in a 24-well plate for 24 h. RNA was isolated and RT–qPCR was performed as previously described. For CD45 selection, tumour tissues were first digested by 1 mg ml^−1^ collagenase IV and 0.2 mg ml^−1^ DNase I (Sigma-Aldrich) for 45 min at 37 °C, then passed through a 70 μm nylon cell strainer to obtain single cells. CD45^+^ leukocytes and CD45^−^ cell populations were collected using magnetic separation using CD45 TIL microbeads and MS columns (Miltenyi Biotec) according to the manufacturer’s instructions before RT–qPCR analysis.

### Reporting Summary

Further information on research design is available in the Nature Research Reporting Summary linked to this article.

## Supplementary information

Supplementary Information Supplementary Figs. 1–14 and Table 1.

Reporting Summary

Peer Review File

## Data Availability

The main data supporting the results in this study are available within the paper and its Supplementary Information. All data generated in this study, including source data and the data used to generate the figures, are available at figshare (10.6084/m9.figshare.13356464).
