# Author Correction: Prolonged activation of innate immune pathways by a polyvalent STING agonist

**DOI:** 10.1038/s41551-021-00741-w

**Published:** 2021-05-07

**Authors:** Suxin Li, Min Luo, Zhaohui Wang, Qiang Feng, Jonathan Wilhelm, Xu Wang, Wei Li, Jian Wang, Agnieszka Cholka, Yang-xin Fu, Baran D. Sumer, Hongtao Yu, Jinming Gao

**Affiliations:** 1 Department of Pharmacology, Harold C. Simmons Comprehensive Cancer Center, University of Texas Southwestern Medical Center, Dallas, TX USA; 2 Department of Pathology, Harold C. Simmons Comprehensive Cancer Center, University of Texas Southwestern Medical Center, Dallas, TX USA; 3 Department of Otolaryngology, Harold C. Simmons Comprehensive Cancer Center, University of Texas Southwestern Medical Center, Dallas, TX USA; 4 Howard Hughes Medical Institute, University of Texas Southwestern Medical Center, Dallas, TX USA; 5 Present Address: Zhejiang Provincial Laboratory of Life Sciences and Biomedicine, School of Life Sciences, Westlake University, Hangzhou, China

**Keywords:** Cancer, Cancer therapy, Biomaterials

Correction to: *Nature Biomedical Engineering* 10.1038/s41551-020-00675-9, published online 8 February 2021.

This Article was originally published online incorrectly without open access, whereas it is actually covered by an Open Access licence (CC BY 4.0). The copyright line in the PDF mistakenly read “© The Author(s), under an exclusive licence to Springer Nature Limited 2021” but should have been ‘© The Author(s) 2021’.

