# Supplementary material for: Prolonged activation of innate immune pathways by a polyvalent STING agonist
Source: Nat Biomed Eng. 2021 Feb 8;5(5):455–66. doi: 10.1038/s41551-020-00675-9 (PMC8126516; doi:10.1038/s41551-020-00675-9)

nature portfolio

https://doi.org/10.1038/s41551-020-00675-9

Supplementary information

Prolonged activation of innate immune pathways by a polyvalent STING agonist

In the format provided by the
authors and unedited

Table of contents

Supplementary Fig. 1 | PC7A polymer induces durable immune activation and prevents rapid STING degradation compared to cGAMP........2

Supplementary Fig. 2 | PC7A polymer activates STING through ER-ERGIC-Golgi translocation..........3

Supplementary Fig. 3 | PC7A polymer shows STING-specific binding affinity, phase condensation, and immune activation compared to PEPA and other polymers........4

Supplementary Fig. 4 | PC7A polymer induces STING oligomerization and condensation in which the two species express different recovery kinetics........5

Supplementary Fig. 5 | Longer PC7A chain length induces larger condensate formation and slower recovery of STING........6

Supplementary Fig. 6 | High salt and non-specific protein concentrations hinder STING-PC7A condensation........7

Supplementary Fig. 7 | PC7A polymer activates STING through a different binding site from cGAMP. 8

Supplementary Fig. 8 | Characterization of cGAMP-loaded PC7A NPs........9

Supplementary Fig. 9 | cGAMP-loaded PC7A NPs achieves both rapid and sustained ifn-β and cxcl10 expressions........10

Supplementary Fig. 10 | Evaluation of antitumor efficacy and safety of different doses of free cGAMP and comparison with cGAMP-PC7A NP........11

Supplementary Fig. 11 | cGAMP-PC7A NP synergizes with anti-PD1 in immunotherapy of tumor-bearing animals........12

Supplementary Fig. 12 | Evaluation of STING status and immune cell type on PC7A NP-induced antitumor immunity........13

Supplementary Fig. 13 | PC7A and cGAMP show synergistic effect in immune activation in additional human tumor tissues........14

Supplementary Fig. 14 | Diagram of PC7A-induced STING phase condensation and immune activation........15

Supplementary Table 1 | Mutation of $E^{296}A/D^{297}A$ in STING abolishes its affinity to PC7A........16

Uncropped Original Scans........17

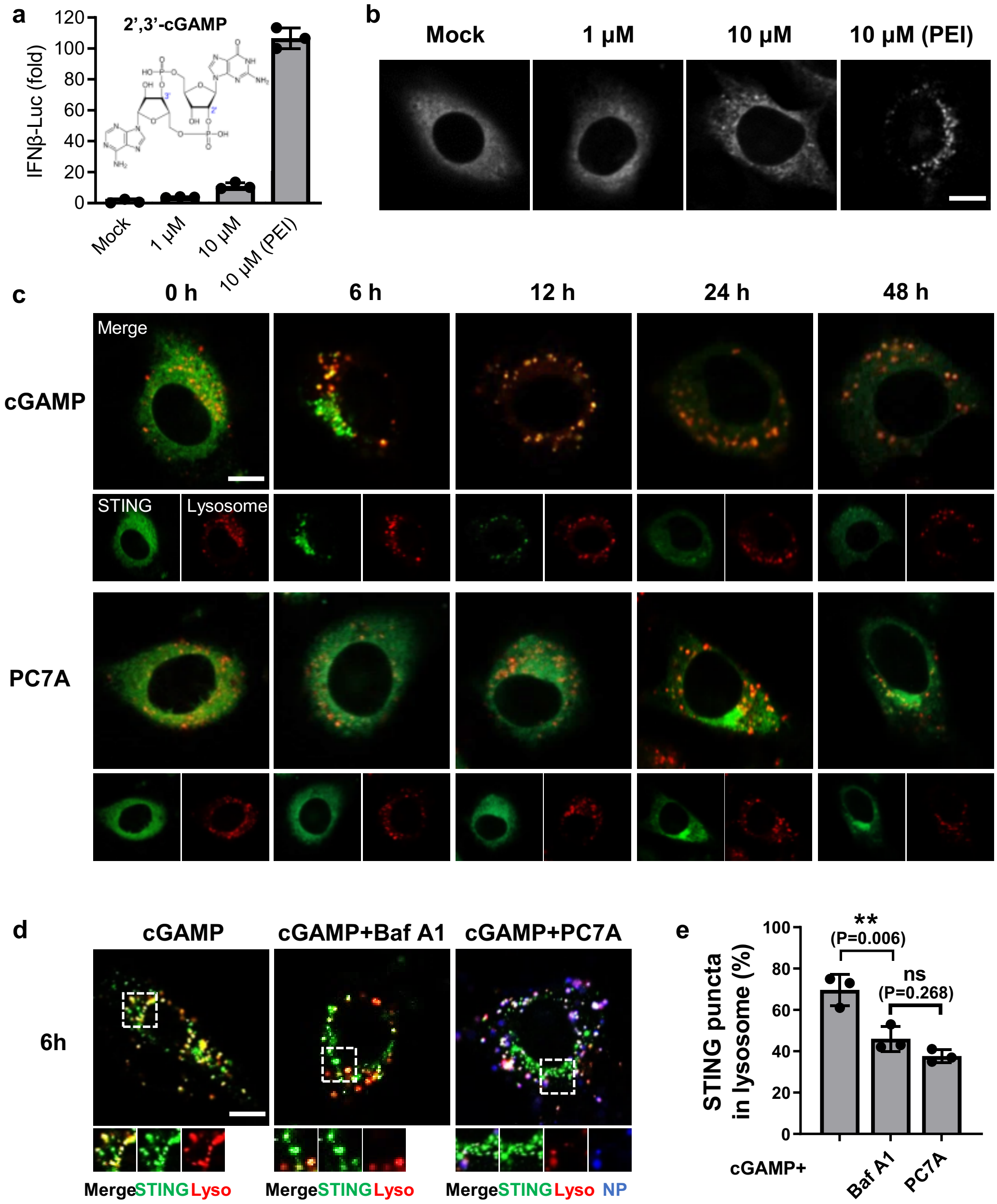

Supplementary Fig. 1 | PC7A polymer induces durable immune activation and prevents rapid STING degradation compared to cGAMP. a, b, Free cGAMP alone has limited STING activation in ISG-THP1 **(a)** and STING-GFP MEF cells **(b)** due to limited membrane permeability. A transfection agent, polyethyleneimine or PEI, was used to aid cytosolic delivery of cGAMP in the ensuing studies unless stated otherwise. **c,** STING proteins rapidly degrade within 12 h after cGAMP treatment, whereas PC7A prevents STING degradation over 48 h. Confocal microscopy images show varying degrees of colocalization of STING-GFP and lysosomes over time after cGAMP or PC7A treatment in MEFs. STING-GFP is shown in green and lysosomes were stained with LysoTracker DND-99 shown in red. **d, e,** Treatment by PC7A or Bafilomycin A1 (Baf A1) reduced the fusion of cGAMP-induced STING puncta with lysosomes. In experiments **a** and **e**, values are mean ± SD, n=3 biologically independent experiments. *One-way ANOVA*. Confocal images in **b-d** are representative of at least three biologically independent experiments, scale bars, 10 μm.

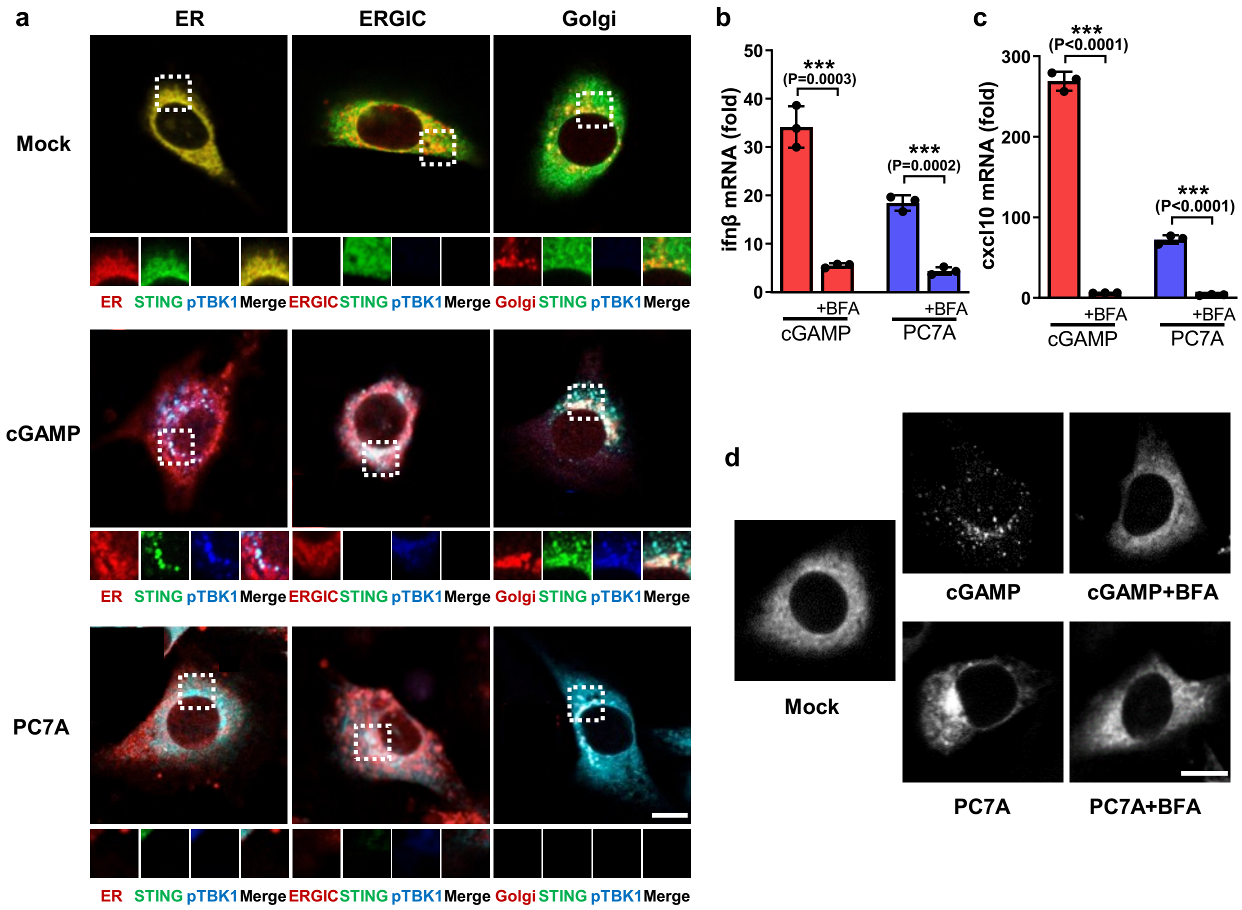

Supplementary Fig. 2 | PC7A polymer activates STING through ER-ERGIC-Golgi translocation. a, STING-GFP is co-localized with ERGIC and Golgi following treatment by cGAMP or PC7A. STING-GFP MEF cells were first incubated with PEI-cGAMP (10 μM) or PC7A micelles (10 μM) for 1 h, followed by media exchange. Cells in cGAMP and PC7A treatment groups were fixed 6 h and 24 h later, respectively, prior to staining p-TBK1, ER (Calnexin), ERGIC (P58), Golgi (GM130), or nucleus. **b-d,** Brefeldin A (BFA) abolishes cGAMP or PC7A-induced STING activation in THP1 **(b, c)** and STING-GFP MEF **(d)** cells. In inhibited groups, cells were pre-treated with BFA (10 μM) before cGAMP or PC7A addition. In experiments **b** and **c**, values are mean ± SD, n=3 biologically independent experiments. *Two-tailed Student's t-test.* Confocal images in **a** and **d** are representative of at least three biologically independent experiments, scale bars, 10 μm.

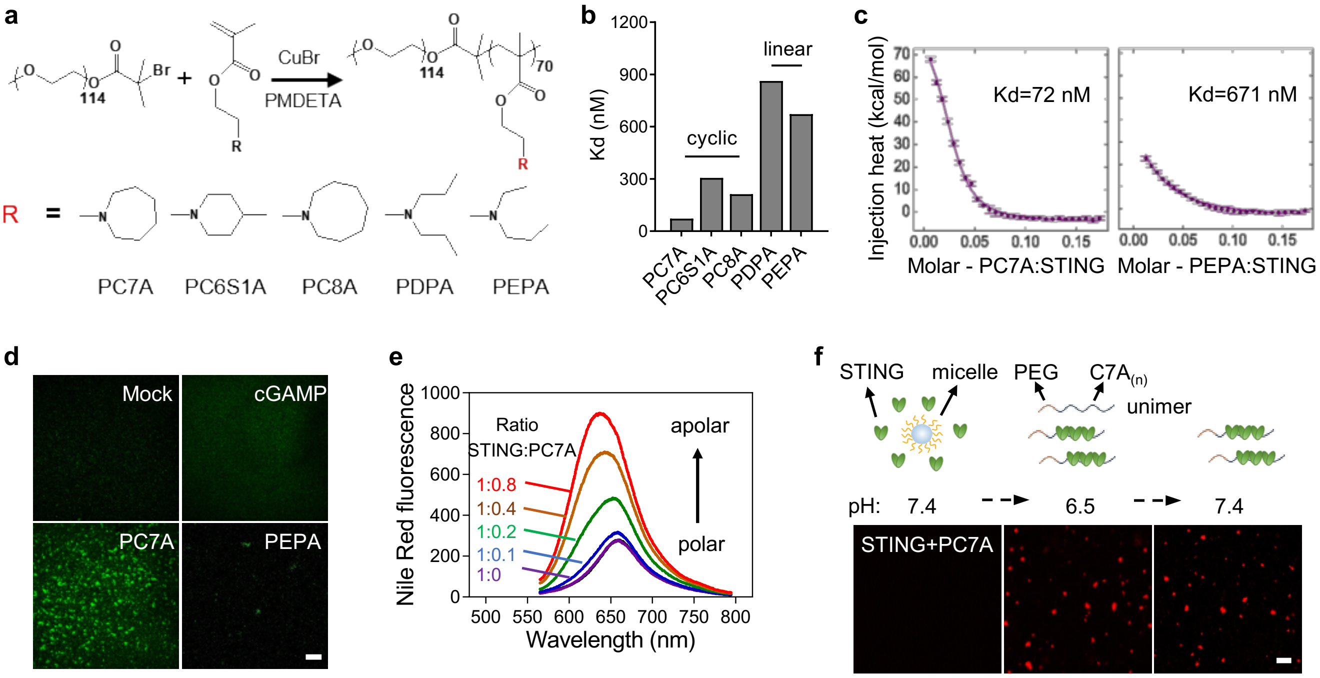

Supplementary Fig. 3 | PC7A polymer shows STING-specific binding affinity, phase condensation, and immune activation compared to PEPA and other polymers. a, Schematic syntheses of block copolymers with different side chain structures using an atom-transfer radical polymerization (ATRP) method. **b,** ITC shows apparent binding affinity between STING and different polymers. Five polymers were divided into two groups based on their cyclic or linear side chains. **c,** PC7A and PEPA, two polymers of the same backbone structure and identical pH transition (6.9), have different binding affinities to STING as measured by ITC. **d,** PC7A, not PEPA or cGAMP, induces STING phase separation from cell lysates. STING-GFP MEF cell lysate was treated with cGAMP, PC7A, or PEPA for 4 h. **e,** Fluorescence spectra of Nile Red in STING solutions with increasing concentrations of PC7A suggests the formation hydrophobic biomolecular condensates. **f,** PC7A tertiary amine blocks are shielded in the hydrophobic core of micelles under neutral pH (7.4), preventing their interactions with STING and phase condensation. Micelles dissociate into cationic unimers at pH 6.5 and induce STING phase separation. The newly formed PC7A-STING condensates are not pH reversible as indicated by the presence of condensates when the pH is titrated back to 7.4. Fluorescent images in **d** and **f** are representative of at least three biologically independent experiments, scale bars, 20 μm.

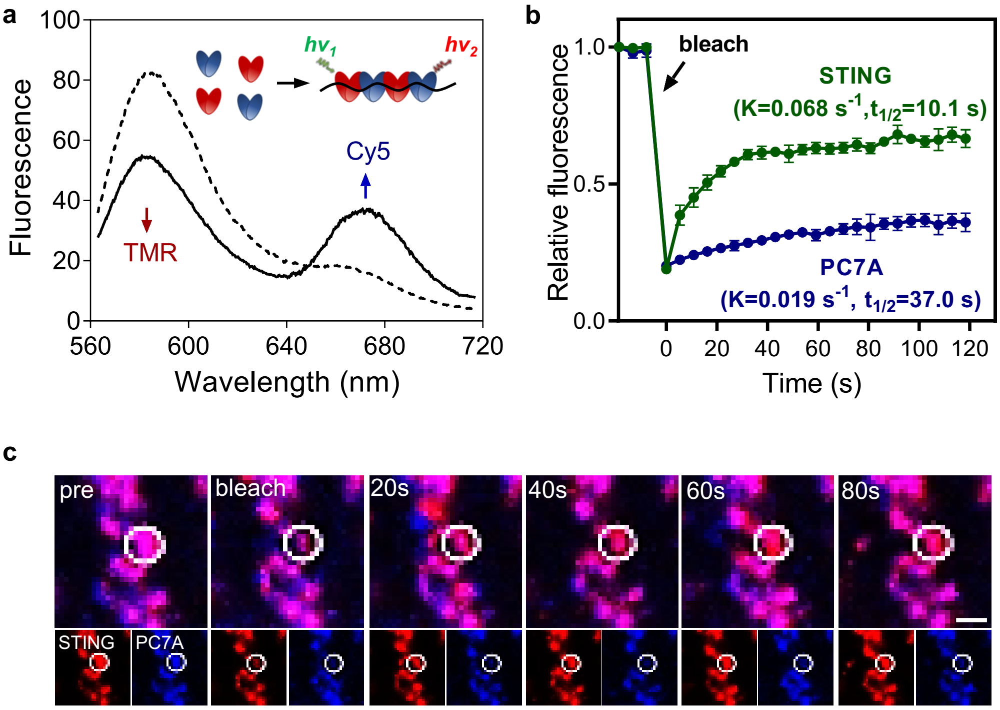

Supplementary Fig. 4 | PC7A polymer induces STING oligomerization and condensation in which the two species express different recovery kinetics. a, Fluorescent spectra show hetero-FRET between STING dimers labeled by a FRET pair, TMR and Cy5 (mixed in a 1:1 ratio) after PC7A treatment (solid line). The decrease of TMR signal and increase of Cy5 signal after PC7A addition indicate STING oligomerization. **b, c,** STING protein or PC7A polymer in condensates exhibits different exchange kinetics by fluorescence recovery after photobleaching (FRAP) measurement. STING (4 µM, Cy5-labeled) and PC7A polymer (2 µM, AMCA-labeled) were incubated for 4 h. After photobleaching, recovery was observed over 120 s. Values are mean ± SD, n=5 cells examined over 2 independent experiments. Fluorescence intensities of regions of interest were fit to the single exponential model: $I_t=I_0+(I_\infty-I_0)\times(1-e^{-kt})$. Scale bar, 2 µm.

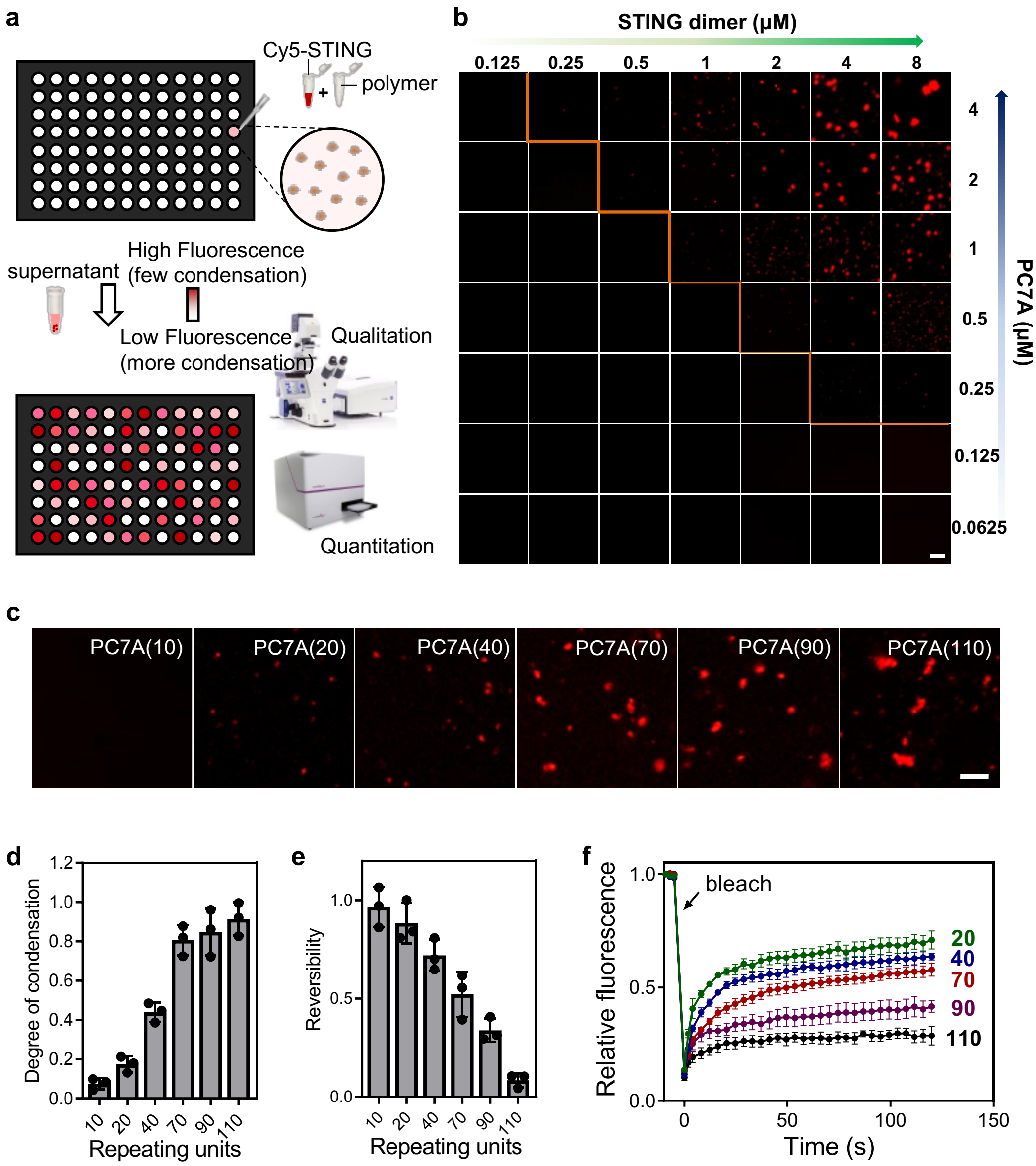

Supplementary Fig. 5 | Longer PC7A chain length induces larger condensate formation and slower recovery of STING. a, Schematic shows the qualitative and quantitative methods used to test the degree of condensation. **b,** Fluorescent images of condensates induced by indicated concentrations of PC7A and STING were used to generate phase diagrams. PC7A(70) is used as an example. **c,** PC7A of higher repeating units induce the formation of larger PC7A-STING condensates. **d,** Degree and **e,** reversibility of STING-PC7A condensates are inversely related. Values are mean ± SD, n=3 biologically independent experiments. **f,** STING recovery rate decreases with increasing PC7A length determined by FRAP method. Values are mean ± SD, n=5 cells examined over 2 independent experiments. In all experiments, STING (4 μM, Cy5-labeled) and PC7A polymer (140 μM C7A modular concentration unless otherwise noted) were incubated for 4 h prior to analysis. Fluorescent images in **b** and **c** are representative of at least three biologically independent experiments, scale bars, 20 μm.

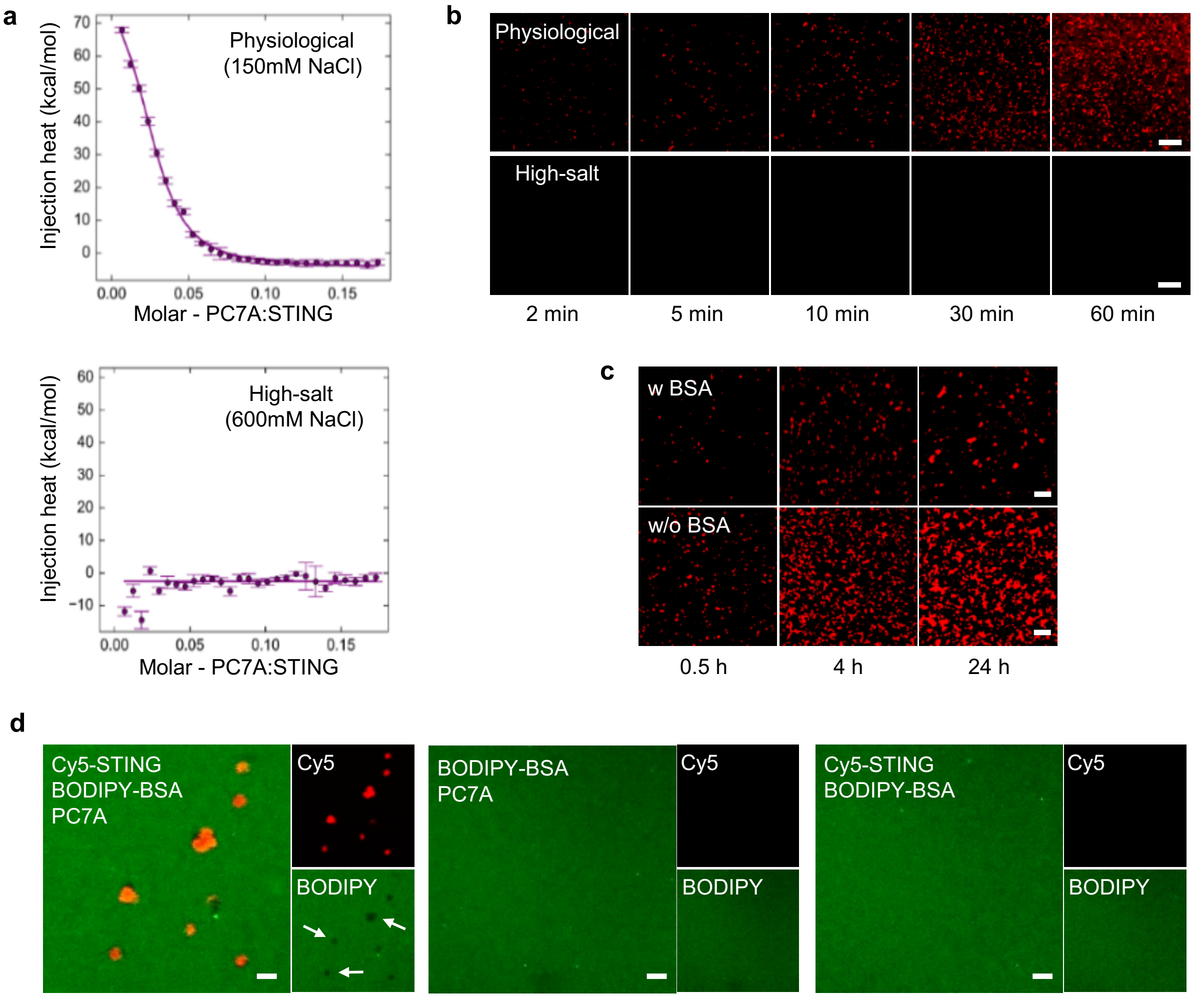

Supplementary Fig. 6 | High salt and non-specific protein concentrations hinder STING-PC7A condensation. a, b, High salt concentration (e.g., 600 mM NaCl) abolishes binding and condensation of PC7A-STING. Scale bar, 20 μm. **c,** PC7A-STING condensates decrease in number and size in the presence of bovine serum albumin (BSA). Scale bar, 20 μm. **d,** BSA (labelled by BODIPY) is excluded from PC7A-STING condensates (lack of green fluorescence in the puncta). STING, BSA, and PC7A polymer were mixed for 4 h before observation under a confocal microscope. Controls without STING or PC7A were used to confirm STING-PC7A specificity in condensate formation. Scale bar, 10 μm. Experiments in **b-d** were performed with STING dimer (4 μM, Cy5-labeled) and PC7A (2 μM). BSA or BODIPY-labeled BSA (8 μM) were used. Fluorescent images are representative of at least three biologically independent experiments.

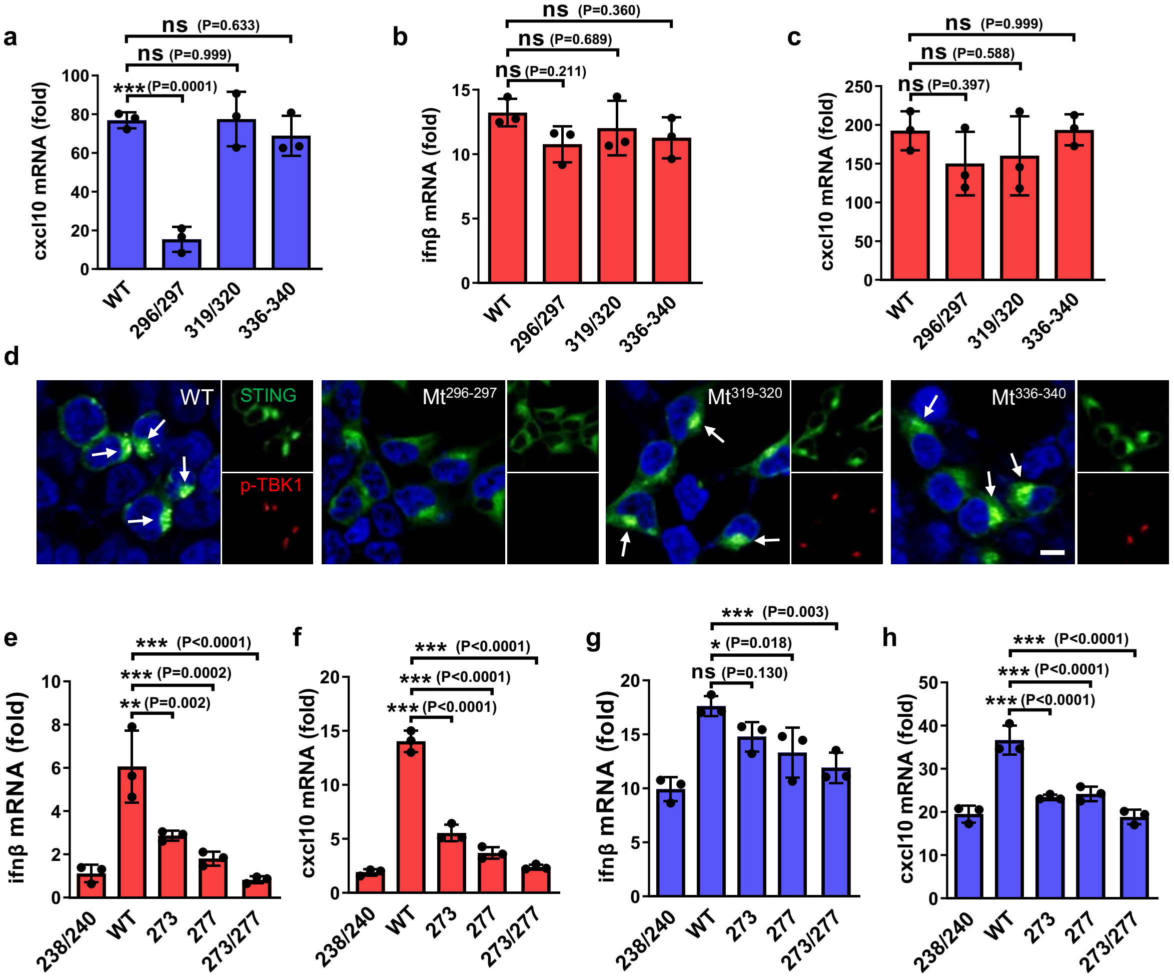

Supplementary Fig. 7 | PC7A polymer activates STING through a different binding site from cGAMP. a-c, Mutation of E296D297 to neutral Ala residues abolishes ifn-b/cxcl10 mRNA expression by PC7A polymer (**a**), but has no effect on cGAMP-stimulated STING response (**b, c**). **d,** Mutation of E296D297 abolishes intracellular p-TBK1 production after PC7A treatment. Confocal images are representative of at least three biologically independent experiments, scale bar, 10 µm. In **a-d**, HEK293T cells were transfected with WT or mutant STING-GFP plasmids for 24 h before use. **e-h,** STING mutant Hela cells (R238A/Y240A, Q273A/A277Q) abolish cGAMP-mediated STING activation (**e, f**), whereas they had less effects on PC7A-mediated response (**g, h**). R238A/Y240A is resistant to cGAMP binding, while single or dual Q273A/A277Q mutation disrupts the tetramer interface of cGAMP-induced STING oligomerization. In each experiment of **a-c** and **e-h**, values are mean ± SD, n=3 biologically independent experiments. *One-way ANOVA.*

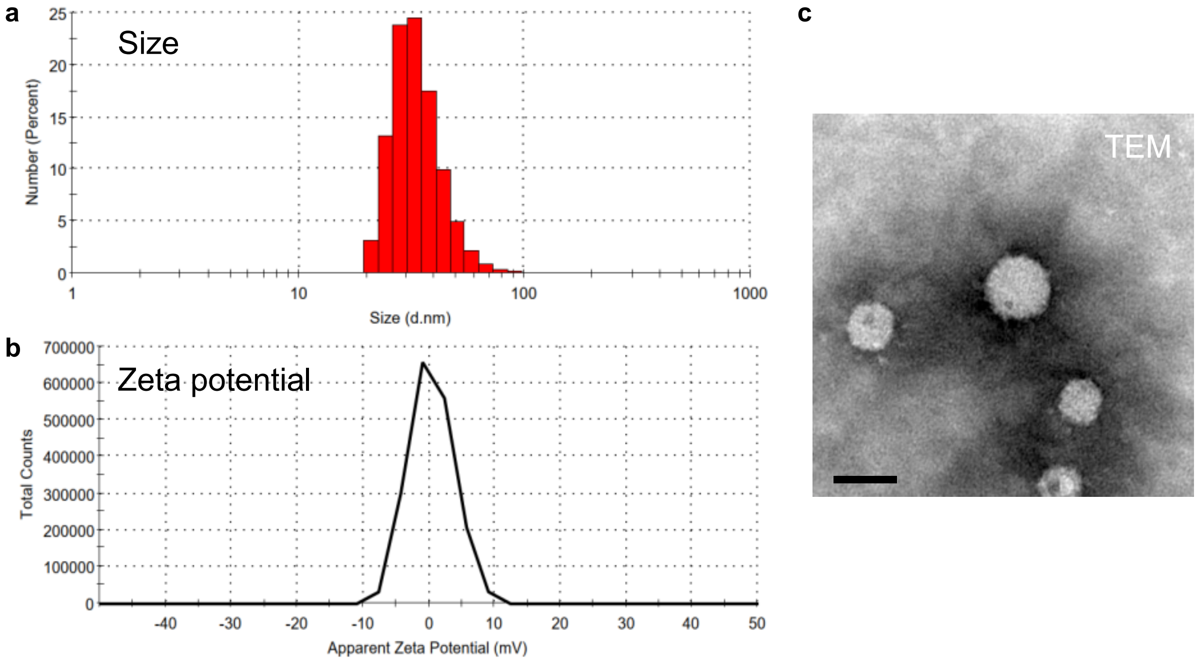

Supplementary Fig. 8 | Characterization of cGAMP-loaded PC7A NPs. Dynamic light scattering analysis was used to measure the hydrodynamic diameter **(a)** and zeta potential **(b)** of cGAMP-loaded PC7A NPs. Transmission electron microscopy (TEM) was used to analyze the size and morphology of the nanoparticles. Scale bar, 50 nm. Data are representative of at least three independent experiments.

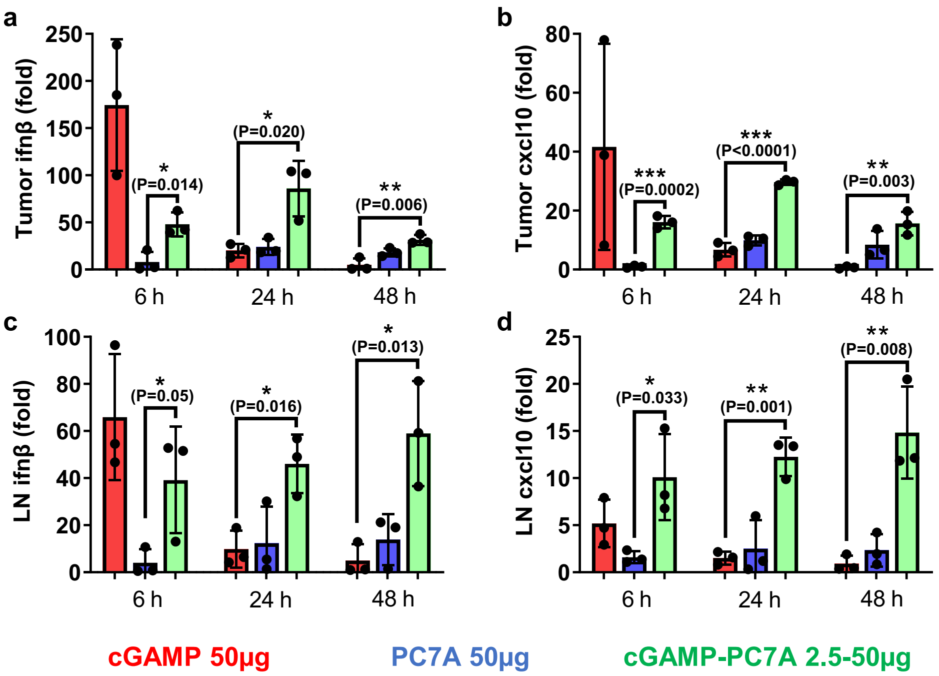

Supplementary Fig. 9 | cGAMP-loaded PC7A NPs achieves both rapid and sustained ifn-β and cxcl10 expressions. Ifn-β and cxcl10 gene expressions in tumor **(a, b)**, or draining lymph node (DLN) **(c, d)** in murine colorectal MC38 mouse model after the indicated treatments. Values are mean ± SD, n=3 biologically independent mous samples. *One-way ANOVA*. Data shows cGAMP-PC7A NP yielded the most optimal STING activity profile with rapid rise of ifn-β/cxcl10 expressions over PC7A at 6 h, but also sustained the activity over 48 h unlike free cGAMP.

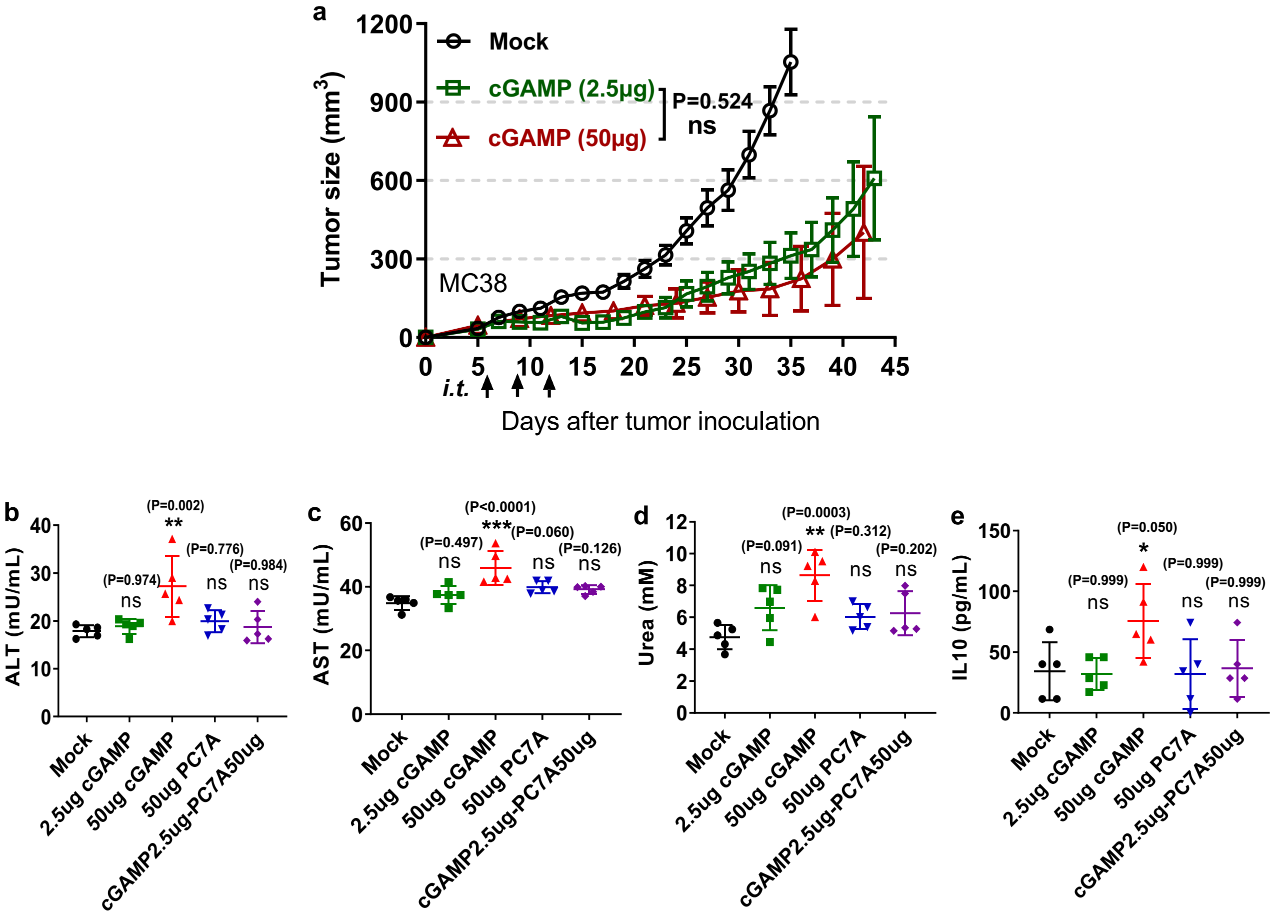

Supplementary Fig. 10 | Evaluation of antitumor efficacy and safety of different doses of free cGAMP and comparison with cGAMP-PC7A NP. a, Tumor growth curves of MC38 tumor-bearing mice injected intratumorally with 5% glucose (mock), low (2.5 μg) or high (50 μg) dose of free cGAMP. Values represent mean ± SEM, n=5 biologically independent mice in each group. *Two-tailed Student's t-test.* **b-e**, High dose of free cGAMP treatment significantly impaired liver (ALT/AST) **(b, c)** and kidney (urea) **(d)** functions and elevated systemic cytokine level (IL10) **(e)**. In contrast, cGAMP-loaded PC7A NP (2.5 μg cGAMP in 50 μg PC7A) group achieved potent tumor growth inhibition (Fig. 5a) without significant increase of immune-related toxicity over the untreated control. In toxicity studies, values are mean ± SD, n=5 biologically independent mouse serum samples in each group. *One-way ANOVA* (versus mock).

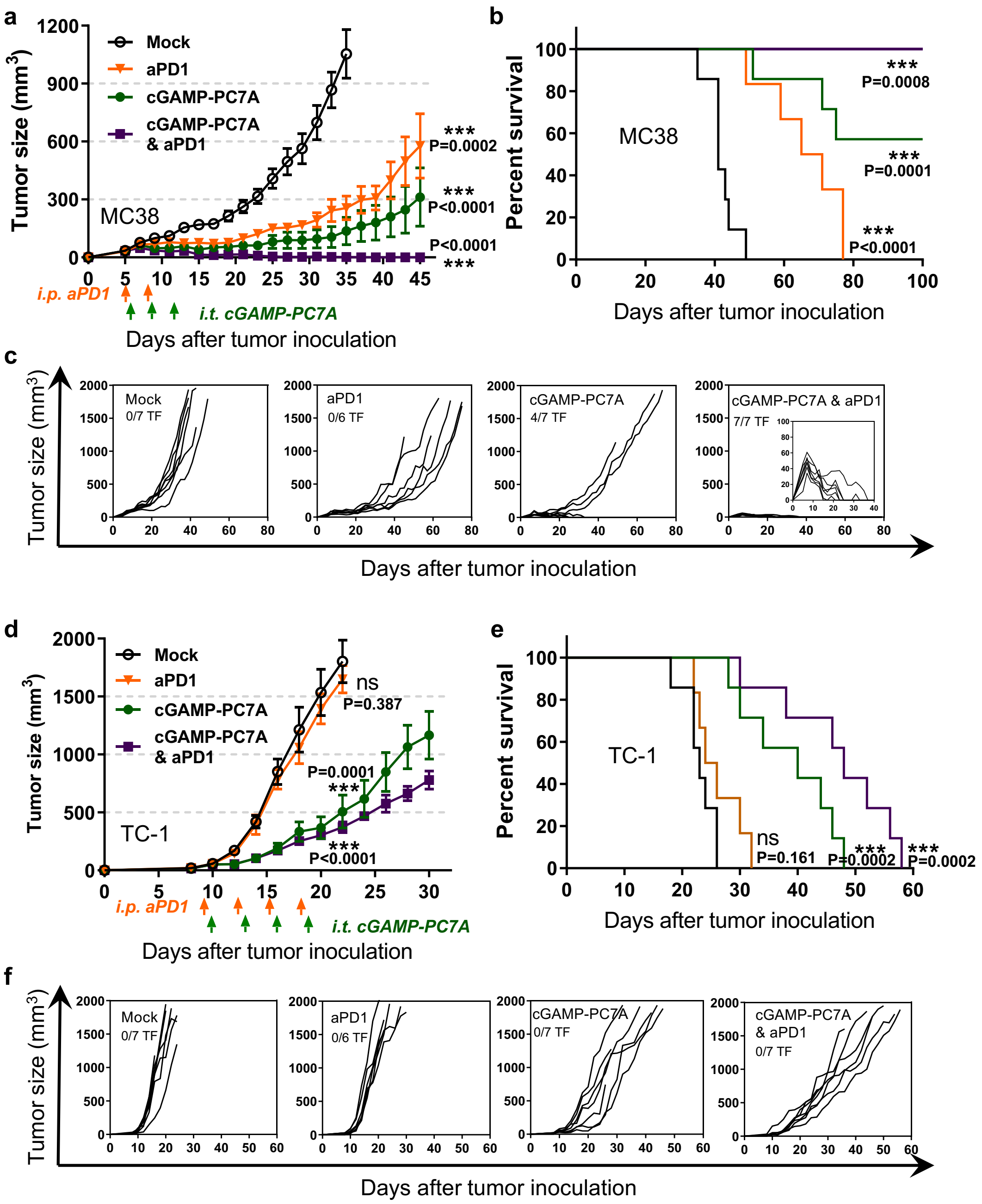

Supplementary Fig. 11 | cGAMP-PC7A NP synergizes with anti-PD1 in immunotherapy of tumor-bearing animals. (a-c) TC1 and **(d-f)** MC38 tumor-bearing mice were injected intratumorally with 5% glucose (mock) or cGAMP-loaded PC7A NP, and injected intraperitoneally with saline or anti-PD1 (200 µg) at indicated time points. Mean tumor volume **(a, d)**, Kaplan–Meier survival curves **(b, e)**, and spider plots of individual tumor growth curves **(c, f)** are shown. cGAMP-PC7A NP treatment confers immune protection, rendering 4/7 MC38 mice tumor free, and further synergizes with anti-PD1 to achieve 100% cure rate in the MC38 model. In tumor growth studies, values represent mean ± SEM, n=7 (mock), n=6 (aPD1), n=7 (cGAMP-PC7A), n=7 (cGAMP-PC7A&aPD1) of biologically independent mice in each tumor model, *two-tailed Student's t-test* (versus mock). In survival studies, *Mantel–Cox test.*

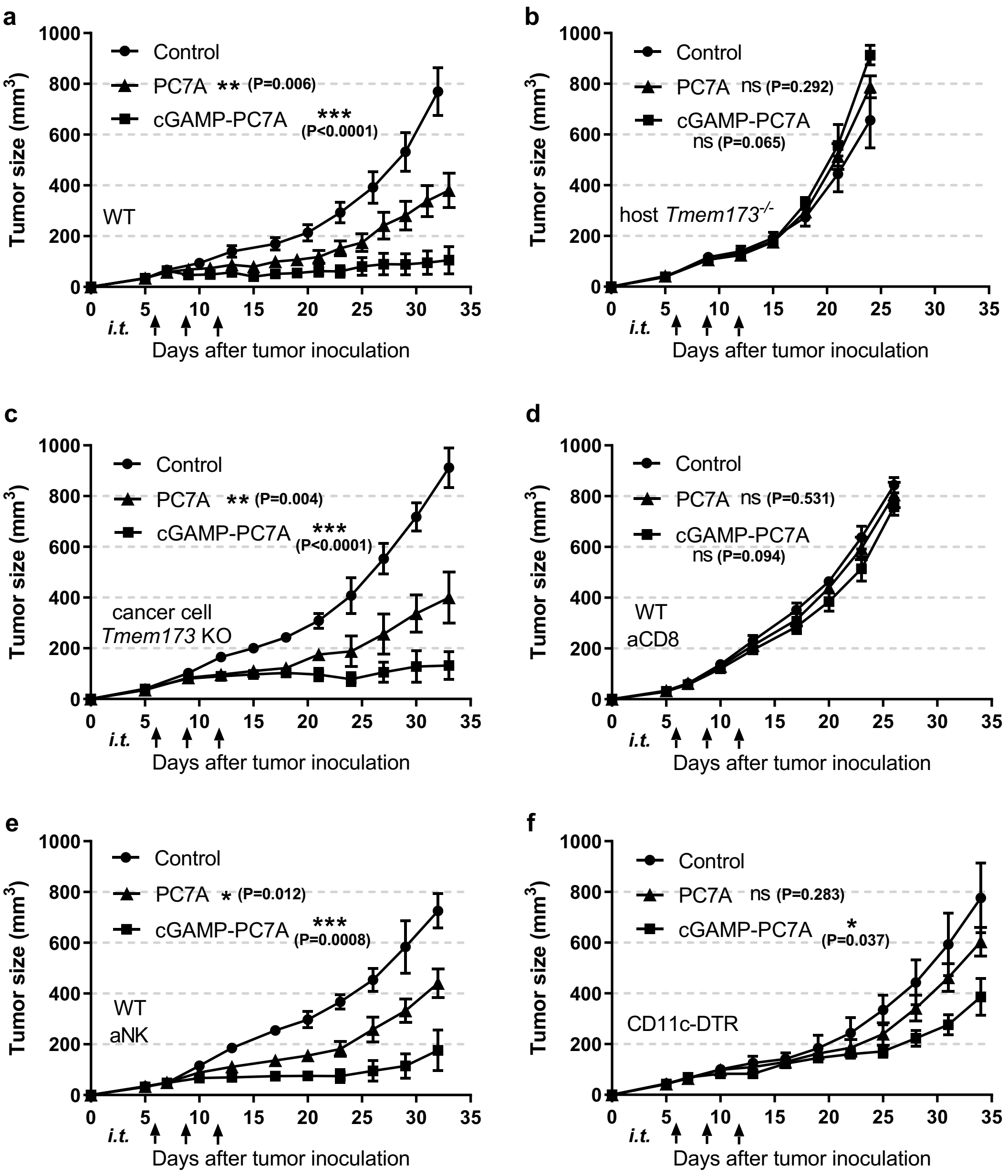

Supplementary Fig. 12 | Evaluation of STING status and immune cell type on PC7A NP-induced antitumor immunity. In each animal cohort, mice were treated with 5% glucose (control), PC7A NP (50 µg), or cGAMP-loaded PC7A NP (2.5 µg cGAMP in 50 µg PC7A). **a-c**, Treatment by PC7A NP and cGAMP-loaded PC7A NP showed completely blocked antitumor efficacy in host *Tmem173*$^{-/-}$ (*Tmem173* encodes STING) mice with WT MC38 cells **(b)**, but not in WT mice with *Tmem173-KO* MC38 cells **(c)**. **d-f**, Tumor growth curves were also measured in wild type mice (inoculated with wild type MC38 cells) with anti-CD8 blockade **(d)**, and with anti-NK1.1 blockade **(e)**, or in CD11c-DTR transgenic mice **(f)** without the dendritic cells. Blockade of CD8 T cells completely abolished the antitumor efficacy whereas blockade of NK cells showed minimal effect. DC depletion in CD11c-DTR mice also impaired the therapeutic outcomes but to a less extent compared to CD8 T blockade. Values represent mean ± SEM, n=5 biologically independent mice in each tumor model. *Two-tailed* S*tudent's t-test* (versus control).

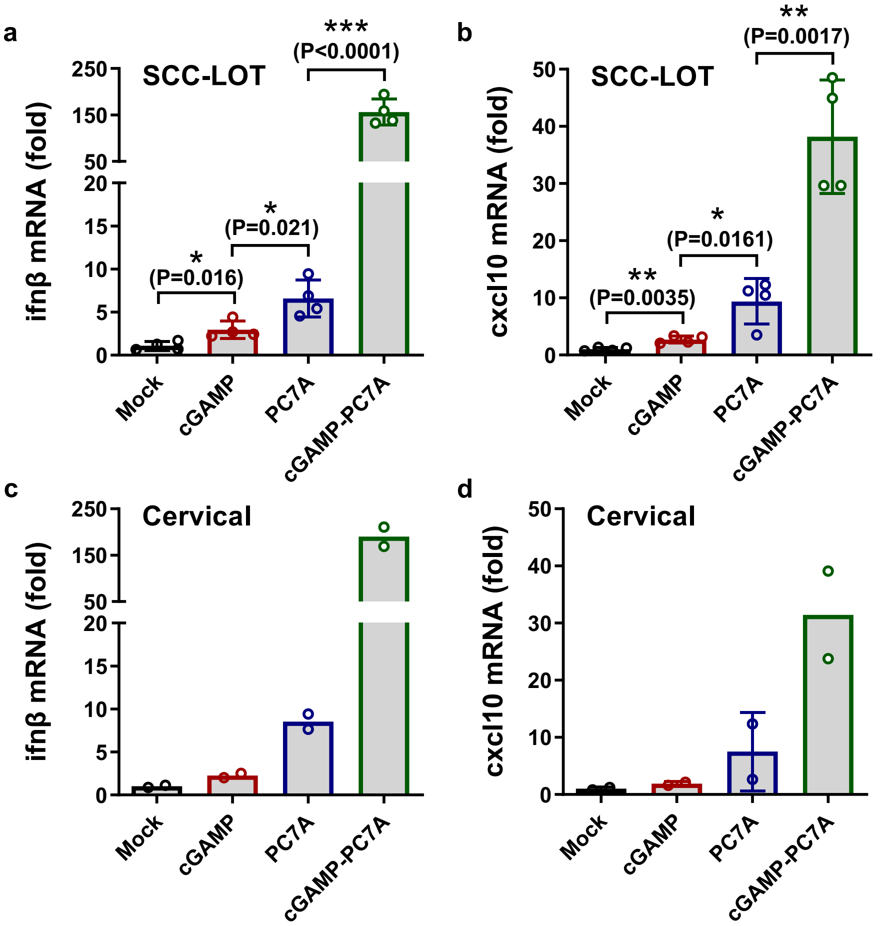

Supplementary Fig. 13 | PC7A and cGAMP show synergistic effect in immune activation in additional human tumor tissues. Ifn-β and cxcl10 gene expression in fresh surgically resected squamous cell carcinoma from the lateral of tongue (SCC-LOT) **(a, b)** and cervical tumor tissues **(c, d)** after injection of 5% glucose, free cGAMP (80 ng), PC7A NPs (50 µg), or cGAMP-loaded PC7A NPs in 5% glucose solution. Values are mean ± SD, n=4 tissue sections from the same SCC-LOT patient in **a** and **b**. *Two-tailed Student's t-test.* n=2 tissue sections from the same cervical cancer patient in **c** and **d.**

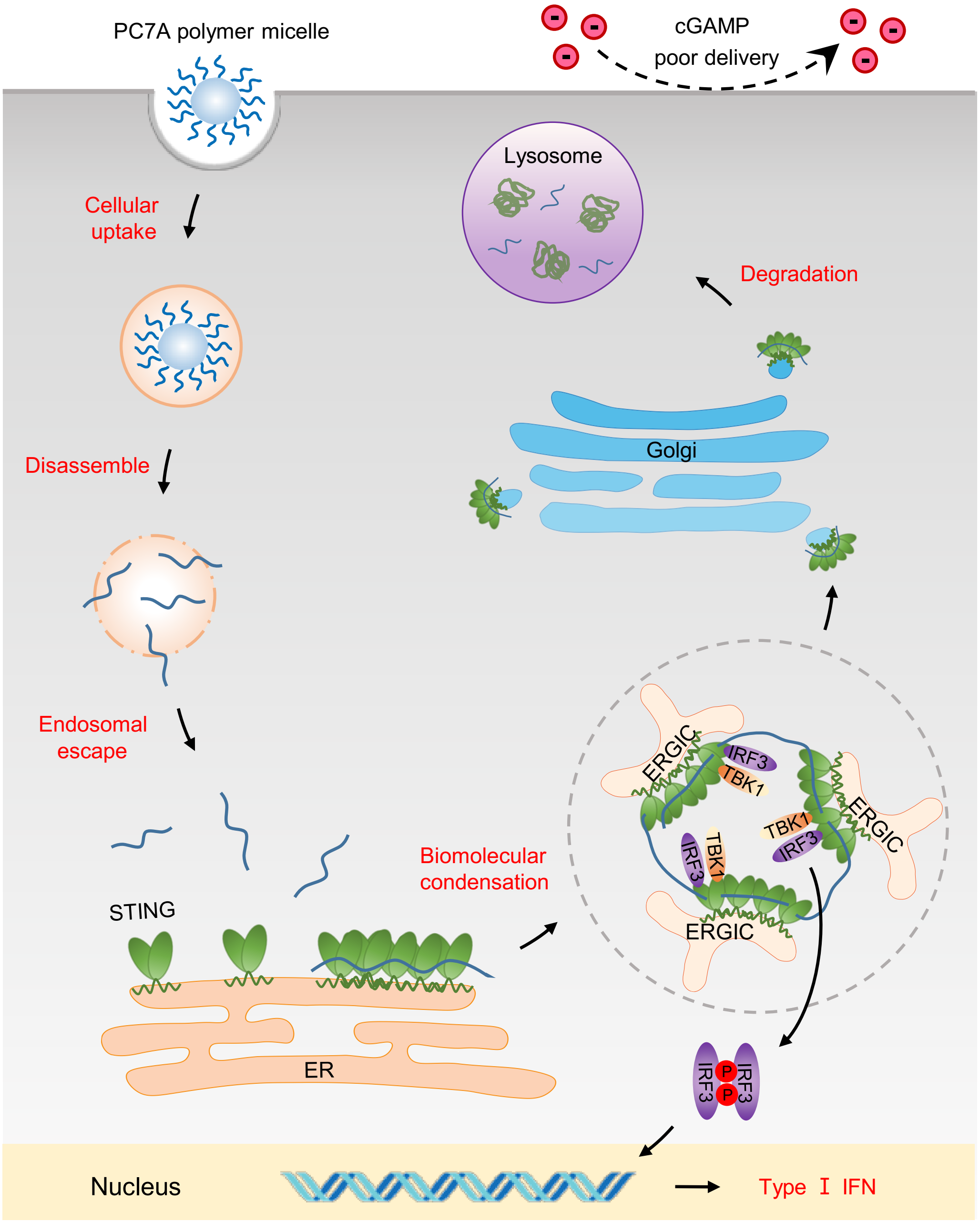

Supplementary Fig. 14 | Diagram of PC7A-induced STING phase condensation and immune activation. PC7A NP enter cells through endocytosis while dual negatively charged cGAMP molecules have limited cell permeability. Upon endosomal maturation and acidification below pH 6.9, PC7A NP disassembles into cationic unimers and escape from endo-lysosomes. In the cytosol, PC7A unimers bind to multiple STING molecules leading to STING oligomerization and condensation during translocation from ER to the ER-Golgi intermediate compartment (ERGIC) and the Golgi apparatus. In the process, STING condensates recruit and trigger the TBK1-IRF3 transcription cascade, leading to the production of type I interferons (IFN) and other proinflammatory cytokines. The activated STING is eventually transported to lysosomes for degradation.

Supplementary Table 1 | Mutation of $E^{296}A/D^{297}A$ in STING abolishes its affinity to PC7A.

STING	Kd
WT	72 nM
$E^{296}A-D^{297}A$	N/A
$D^{319}A-D^{320}A$	147 nM
$E^{336}A-E^{337}A-E^{339}A-E^{340}A$	83 nM

Uncropped Original Scans

Figure 1b

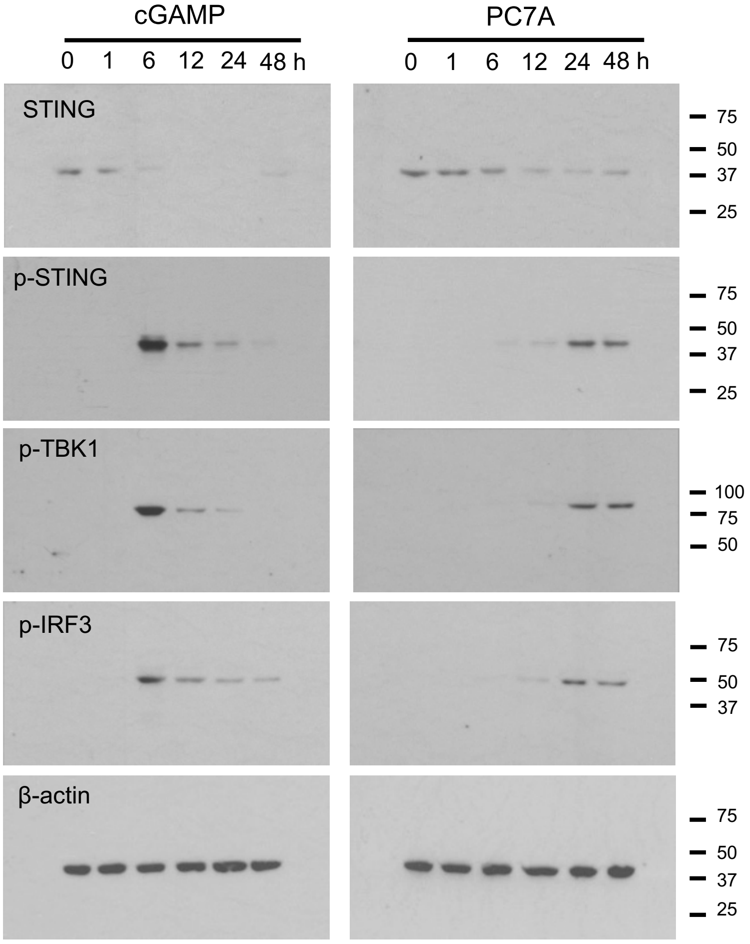

Figure 1f

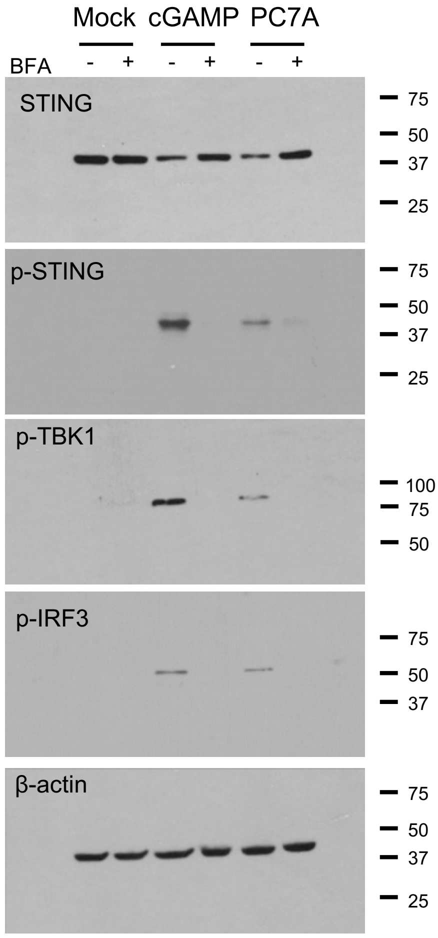

Figure 4f

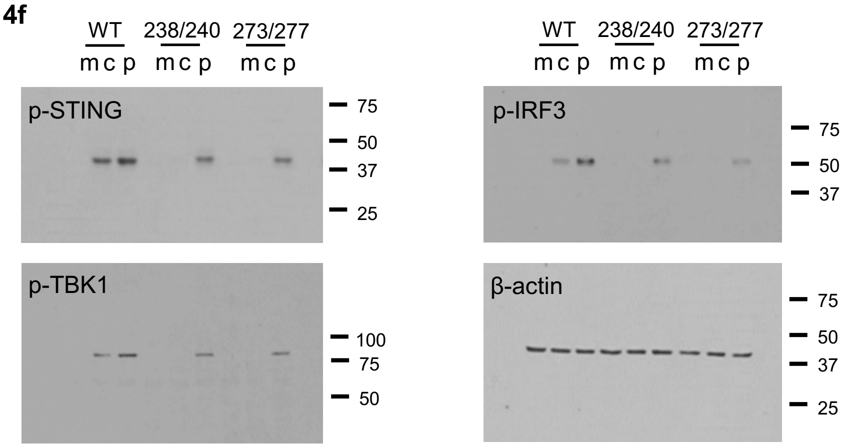

Supplement: Supplementary file 1 — Supplementary Figs. 1–14 and Table 1. [file 41551_2020_675_MOESM1_ESM.pdf]
