# Supplementary material for: Prolonged activation of innate immune pathways by a polyvalent STING agonist
Source: Nat Biomed Eng. 2021 Feb 8;5(5):455–66. doi: 10.1038/s41551-020-00675-9 (PMC8126516; doi:10.1038/s41551-020-00675-9)
Supplement: Supplementary file 2 — Reporting Summary [file 41551_2020_675_MOESM2_ESM.pdf]

nature research

Corresponding author(s): Jinming Gao

Last updated by author(s): Nov 4, 2020

Reporting Summary

Nature Research wishes to improve the reproducibility of the work that we publish. This form provides structure for consistency and transparency in reporting. For further information on Nature Research policies, see Authors & Referees and the Editorial Policy Checklist.

Statistics

For all statistical analyses, confirm that the following items are present in the figure legend, table legend, main text, or Methods section.

n/a	Confirmed	
☐	☒	The exact sample size (*n*) for each experimental group/condition, given as a discrete number and unit of measurement
☐	☒	A statement on whether measurements were taken from distinct samples or whether the same sample was measured repeatedly
☐	☒	The statistical test(s) used AND whether they are one- or two-sided<br>*Only common tests should be described solely by name; describe more complex techniques in the Methods section.*
☒	☐	A description of all covariates tested
☒	☐	A description of any assumptions or corrections, such as tests of normality and adjustment for multiple comparisons
☐	☒	A full description of the statistical parameters including central tendency (e.g. means) or other basic estimates (e.g. regression coefficient) AND variation (e.g. standard deviation) or associated estimates of uncertainty (e.g. confidence intervals)
☐	☒	For null hypothesis testing, the test statistic (e.g. *F*, *t*, *r*) with confidence intervals, effect sizes, degrees of freedom and *P* value noted<br>*Give P values as exact values whenever suitable.*
☒	☐	For Bayesian analysis, information on the choice of priors and Markov chain Monte Carlo settings
☒	☐	For hierarchical and complex designs, identification of the appropriate level for tests and full reporting of outcomes
☐	☒	Estimates of effect sizes (e.g. Cohen's *d*, Pearson's *r*), indicating how they were calculated

Our web collection on statistics for biologists contains articles on many of the points above.

Software and code

Policy information about availability of computer code

Data collection	Confocal images were taken with the built-in software (ZEN 2.6) of the Zeiss 700 confocal laser scanning microscope. mRNA expressions were measured by the Bio-Rad CFX Connect Real-Time PCR Detection System. Luciferase activities and fluorescent intensity of phase separation assays were measured with the CLARIOstar plate reader. Titration curves were generated with the MicroCal VP-ITC system.
Data analysis	Statistical analyses were performed with Graph Pad Prism 7 and Excel Office 365. For co-localization analyses, the Pearson's correlation coefficient was calculated via ImageJ 1.52d. For the ITC assay, titration traces were integrated by NITPIC 1.2.7, the curves were fitted by SEDPHAT 15.2b, and the figures were prepared by using GUSSI 1.4.2 software.

For manuscripts utilizing custom algorithms or software that are central to the research but not yet described in published literature, software must be made available to editors/reviewers. We strongly encourage code deposition in a community repository (e.g. GitHub). See the Nature Research guidelines for submitting code & software for further information.

Data

Policy information about availability of data

All manuscripts must include a data availability statement. This statement should provide the following information, where applicable:

- Accession codes, unique identifiers, or web links for publicly available datasets
- A list of figures that have associated raw data
- A description of any restrictions on data availability

The main data supporting the results in this study are available within the paper and its Supplementary Information. All data generated in this study, including source data and the data used to generate the figures, are available from figshare with the identifier https://doi.org/10.6084/m9.figshare.13356464.

Field-specific reporting

Please select the one below that is the best fit for your research. If you are not sure, read the appropriate sections before making your selection.

☒ Life sciences ☐ Behavioural & social sciences ☐ Ecological, evolutionary & environmental sciences

For a reference copy of the document with all sections, see nature.com/documents/nr-reporting-summary-flat.pdf

Life sciences study design

All studies must disclose on these points even when the disclosure is negative.

Sample size	For RT-PCR, luciferase activity, and quantification of the degree of phase separation, each sample was performed three times independently. For co-localization analyses, 20 confocal images were randomly taken and the Pearson's correlation coefficient was calculated from all the cells in these images. For quantification of STING puncta in lysosomes, 100 puncta were randomly taken from 20 cells and this process was repeated three times. For the FRAP assay, five condensates were randomly picked, and bleaching was performed at the central site of each condensate. For the quantification of condensate size, 50 condensates were randomly taken and their size was calculated as the average of the longest axis and the shortest axis. For the in vivo studies, animals in each model were randomly divided with 5–7 mice per group. For the human-tissue assay, each SLN or SCC-BOT tissue was divided into 16 sections before treatment (4 in each group); the cervical tumor tissue was divided into 8 sections because of their small size (2 in each group). The sample sizes were selected on the basis of a power analysis of results from preliminary experiments, and were consistent with those used in similar reports in the literature, which indicates that the sample sizes are not only sufficient to obtain desirable significance level (< 0.01) and power (> 90%), but also able to generate highly reproducible results with biological replicates.
Data exclusions	No data were excluded.
Replication	RT-PCR and luciferase-activity measurements were performed three times, biologically independently. Imaging experiments (that is, confocal and western blot) were performed at least three times biologically independently, with all replicates generating similar results. In the in vivo studies, 5–7 mice per group were used and no replicates were performed. In human-tissue assays, two technical replicates were performed for each section.
Randomization	For the quantification of condensates or cells with phenotype of interest, images were randomly taken throughout the chamber of each sample. For in vitro cell-based RT-PCR, luciferase-activity measurement, and western-blot assay, all cells under well-controlled conditions were analysed equally; therefore, no randomization was necessary. For the animal study and the human-tissue assay, animals and divided sections were randomly allocated into each group.
Blinding	In all phase-separation assays and cell-based experiments, data collection and analysis were performed in a blinded manner because each sample was only identified with a number that didn't show any information about the treatment administrated. In the animal studies, true blinding was not possible for the administration of treatment owing to practical reasons, but the appropriate controls were present. The human-tissue assay was performed by independent researchers who were blinded as to treatment-group assignment.

Reporting for specific materials, systems and methods

We require information from authors about some types of materials, experimental systems and methods used in many studies. Here, indicate whether each material, system or method listed is relevant to your study. If you are not sure if a list item applies to your research, read the appropriate section before selecting a response.

Materials & experimental systems

n/a	Involved in the study	
☐	☒	Antibodies
☐	☒	Eukaryotic cell lines
☒	☐	Palaeontology
☐	☒	Animals and other organisms
☐	☒	Human research participants
☒	☐	Clinical data

Methods

n/a	Involved in the study	
☒	☐	ChIP-seq
☒	☐	Flow cytometry
☒	☐	MRI-based neuroimaging

Antibodies

Antibodies used	Rabbit Monoclonal anti-STING (13647), Rabbit Monoclonal anti-Phospho-STING (S366) (19781), Rabbit Monoclonal anti-Phospho-TBK1/NAK (Ser172) (5483), and Rabbit Monoclonal anti-Phospho-IRF-3 (Ser396) (4947) were purchased from Cell Signaling. Mouse Monoclonal anti-β-actin (A5441) and Rabbit Polyclonal anti-ERGIC-53/p58 - Cy3 (E6782) were purchased from Sigma Aldrich. Mouse Monoclonal anti-Calnexin - AF647 (ab202572) was from Abcam. Mouse Monoclonal anti-GM130 - AF647 (558712) was from BD Biosciences. Goat Anti-Mouse IgG (H+L) - HRP (1721011) and Goat Anti-Rabbit IgG (H+L) - HRP (1706515) were from Bio-Rad. Anti-mouse PD-1 (BE0146), CD8α (BP0117), and NK1.1 (BP0036) were from Bio X Cell.

Validation	All antibodies are commercially available. Each antibody was validated for species and application, as appropriate, according to the manufacturer's website, and as supported by relevant citations on their product pages.

Eukaryotic cell lines

Policy information about cell lines

Cell line source(s)	The cell lines, including HEK293T, Hela, THP1, B16 and MC38 cells, were obtained from ATCC. WT and R232H STING reporter cells were abtained from InvivoGen. ISG-THP1 cells, R238A/Y240A and single or dual Q273A/A277Q Hela mutants cells were constructed and provided by Z.J.C. (Nature 567.7748 (2019): 394–398). STING-GFP MEF cells were provided by N.Y. (Cell host & microbe 18.2 (2015): 157–168). Tmem173-KO MC38 cells were provided by Y-X. F. (Nature immunology 21.5 (2020): 546–554). TC-1 cells were provided by T. C. Wu (John Hopkins University). ISG-THP1 cells are commercially available from InvivoGen and TC-1 cells are commercially available from ATCC.
Authentication	HEK293T, Hela, THP1, B16, and MC38 cells were authenticated by ATCC. WT and R232H STING reporter cells were authenticated by InvivoGen. No further authentication was performed.
Mycoplasma contamination	The cell lines used in this study were free of mycoplasma contamination, according to results from the e-Myco Mycoplasma PCR Detection Kit (Bulldog Bio), and were regularly maintained with Normocin.
Commonly misidentified lines (See ICLAC register)	No commonly misidentified cell lines were used.

Animals and other organisms

Policy information about studies involving animals; ARRIVE guidelines recommended for reporting animal research

Laboratory animals	Six-to-eight week-old female C57Bl/6 mice were purchased from the UT Southwestern breeding core. Tmem173-/- C57BL/6 mice were provided by Y-X. F. CD11c-DTR transgenic C57BL/6 mice were purchased from the Jackson Laboratory. Mice were housed in a barrier facility with a 12-h light/dark cycle and maintained on standard chow (2916 Teklad Global). The temperature range for the housing room is 68–79 ºF (average is around 72 ºF) and the humidity range is 30–50% (average is around 50%).
Wild animals	The study did not involve wild animals.
Field-collected samples	The study did not involve samples collected from the field.
Ethics oversight	The handling of mice and the experiments with them were conducted under federal, state, and local guidelines and with approval from the Institutional Animal Care and Use Committee at the University of Texas Southwestern Medical Center.

Note that full information on the approval of the study protocol must also be provided in the manuscript.

Human research participants

Policy information about studies involving human research participants

Population characteristics	Surgically resected human tissues were obtained from four individual patients. #1. 55-year-old; male; diagnosis: squamous cell carcinoma of the base of tongue, stage I; treatment: adjuvant radiation and no chemotherapy; current status: clinically and radiographically without evidence of disease. #2. 29-year-old; female; diagnosis: squamous cell carcinoma of the right lateral tongue, stage I; treatment: adjuvant radiation and no chemotherapy; current status: no evidence of persistent disease. #3. 56-year-old; female; diagnosis: cervical cancer, stage IVB; treatment: chemotherapy including cisplatin and paclitaxel; current status: remission for one year. #4. 72-year-old; male; diagnosis: squamous cell carcinoma of the left piriform sinus, stage IVA; treatment: adjuvant radiation and cisplatin; current status: no recurrent neck mass or pathologically enlarged lymphadenopathy.
Recruitment	Patients were invited to participate by one of the investigators or a designee of one of the investigators. If they were willing to participate, the investigator or their designee reviewed the consent form and obtained informed consent. All patients with planned surgery were consented to the tissue-collection protocol. Patients were approached in the clinics by study personnel without relation to age, race, gender, disease stage or prior therapies. Tissue collection and formulation injection were performed by independent researchers who were blinded as to treatment-group assignment. There was no self-selection or any other bias.
Ethics oversight	UT Southwestern Institutional Review Board (IRB number 092013-032).

Note that full information on the approval of the study protocol must also be provided in the manuscript.
